# Changes in cGMP Levels Affect the Localization of EGL-4 in AWC in *Caenorhabditis elegans*

**DOI:** 10.1371/journal.pone.0031614

**Published:** 2012-02-03

**Authors:** Damien M. O'Halloran, O. Scott Hamilton, Jin I. Lee, Maria Gallegos, Noelle D. L'Etoile

**Affiliations:** 1 Center for Neuroscience, University of California Davis, Davis, California, United States of America; 2 Department of Psychiatry and Behavioral Sciences, University of California Davis, Sacramento, California, United States of America; 3 Department of Biological Sciences, George Washington University, Washington, D.C., United States of America; 4 Institute for Neuroscience, George Washington University, Washington, D.C., United States of America; 5 Fred Hutchinson Cancer Research Center, Seattle, Washington, United States of America; 6 Department of Biological Sciences, California State University East Bay, Hayward, California, United States of America; Inserm U869, France

## Abstract

The Protein Kinase G, EGL-4, is required within the *C. elegans* AWC sensory neurons to promote olfactory adaptation. After prolonged stimulation of these neurons, EGL-4 translocates from the cytosol to the nuclei of the AWC. This nuclear translocation event is both necessary and sufficient for adaptation of the AWC neuron to odor. A cGMP binding motif within EGL-4 and the Gα protein ODR-3 are both required for this translocation event, while loss of the guanylyl cyclase ODR-1 was shown to result in constitutively nuclear localization of EGL-4. However, the molecular changes that are integrated over time to produce a stably adapted response in the AWC are unknown. Here we show that odor-induced fluctuations in cGMP levels in the adult cilia may be responsible in part for sending EGL-4 into the AWC nucleus to produce long-term adaptation. We found that reductions in cGMP that result from mutations in the genes encoding the cilia-localized guanylyl cyclases ODR-1 and DAF-11 result in constitutively nuclear EGL-4 even in naive animals. Conversely, increases in cGMP levels that result from mutations in cGMP phosphodiesterases block EGL-4 nuclear entry even after prolonged odor exposure. Expression of a single phosphodiesterase in adult, naive animals was sufficient to modestly increase the number of animals with nuclear EGL-4. Further, coincident acute treatment of animals with odor and the phosphodiesterase inhibitor 3-isobutyl-1-methylxanthine (IBMX) decreased the number of animals with nuclear EGL-4. These data suggest that reducing cGMP levels in AWC is necessary and even partially sufficient for nuclear translocation of EGL-4 and adaptation as a result of prolonged odor exposure. Our genetic analysis and chemical treatment of *C. elegans* further indicate that cilia morphology, as defined by fluorescent microscopic observation of the sensory endings, may allow for odor-induced fluctuations in cGMP levels and this fluctuation may be responsible for sending EGL-4 into the AWC nucleus.

## Introduction

In order to effectively shape behavior, neural circuits must integrate sensory information by collating input and placing this input in a context of previous or concurrent sensory information. This modulation of behavior by previous experience or against a background of current input is termed behavioral plasticity. All sensory systems, namely the visual, olfactory, mechanosensory, thermosensory, and auditory systems, are capable of exhibiting neuronal plasticity [1], [2], [3], [4], [5]. The ability to fine-tune sensory responses is an essential survival tool for both vertebrates and invertebrates.

The nematode *C. elegans* is inherently attracted to a variety of volatile odors. Using a pair of head neurons, called the ‘AWC Right’ and ‘AWC Left’, *C. elegans* can sense and seek out attractive odors such as butanone, benzaldehyde, and isoamyl alcohol [6], [7]. Chemosensation in AWC is mediated by the proposed direct binding of odor to seven transmembrane G-protein coupled receptors, which are exclusively localized to the sensory cilia [8], [9]. Intracellularly, these receptors are coupled to heterotrimeric G-proteins, whose α subunit can positively or negatively regulate odortaxis [10]. There are at least four Gα subunits expressed in AWC, these are: ODR-3; GPA-2; GPA-3; and GPA-13 [10], [11], [12]. Odor signaling in AWC is thought to utilize cGMP as a secondary messenger because the receptor guanylyl cyclases DAF-11 [13] and ODR-1 [14], as well as the cGMP gated calcium channels TAX-2 [15] and TAX-4 [16] are all required for proper AWC mediated odortaxis. Each factor has been shown to localize to the AWC sensory cilia. Genetically encoded calcium reporters have revealed that in the absence of odor the AWC exhibits high intracellular calcium levels, and upon acute odor binding, the intracellular calcium levels decrease leading to the hyperpolarization of the AWC neuron [17]. Thus, it is possible that odor decreases cGMP levels which then favor closing of the cGMP-gated channels and hyperpolarization of the AWC neuron. This schema of acute odor binding in AWC shares many similarities with the light response in photoreceptor cells where in the absence of light calcium channels are open exhibiting a ‘dark current’. Upon binding of photons of light, a cGMP phosphodiesterase is activated which reduces cGMP levels causing closure of cyclic nucleotide gated channels, and thus hyperpolarizing the cell [18], [19].

After prolonged stimulation with a particular odor, *C. elegans* will cease to seek out this odor. This decreased odor-attraction is termed odor adaptation [2], [20], [21], [22]. The Protein Kinase G (PKG) EGL-4 has been shown to be necessary for adaptation of the chemosensory response of the AWCs [20], [23], [24]. EGL-4 is required in the AWC at the time of odor exposure for adaptation to all AWC sensed odors [23]. EGL-4 contains a nuclear localization sequence essential for long-lasting (>2 hour) stable adaptation. Nuclear localization of EGL-4 has been shown to be both necessary and sufficient to promote long-term adaptation [25]. Furthermore, we previously demonstrated that the Gα subunit protein ODR-3 [24] as well as the ability of EGL-4 to bind cGMP [25] are both required for proper nuclear entry of EGL-4 after prolonged odor exposure. However, how these signals might be integrated over time to regulate EGL-4 nuclear entry is not known. Previously we published that genetic loss of the guanylyl cyclase ODR-1 led to constitutively nuclear EGL-4 [24] while here we show that decreases in cGMP levels at the time of odor exposure (which we will refer to as “dynamic” changes in order to contrast them with the chronically lower levels of cGMP that the guanylyl cyclase deficient animals are predicted to experience throughout their development) influence the localization of EGL-4. We examined the localization of a GFP tagged EGL-4 molecule in a strain that lacks the guanylyl cyclase DAF-11 [13]. This strain is predicted to have lower levels of cGMP within AWC. We found that in this mutant background GFP::EGL-4 was always in the nucleus of AWC in naive (unexposed) animals. When we examined the localization of GFP::EGL-4 in a cGMP phosphodiesterase (PDE) mutant background [26], where cGMP levels are predicted to be high, we found that EGL-4 failed to translocate to the nucleus of AWC even after prolonged odor-exposure. Each observation of animals that have been genetically modified to alter cGMP levels is consistent with the explanation that odor may induce nuclear translocation of EGL-4 by decreasing cGMP levels. However, the possibility remained that the altered cGMP levels that the animal experienced during development were responsible for changing EGL-4′s subcellular localization in the adult. To distinguish between the possibilities that the lack of PDE activity during development versus in the adult during prolonged odor exposure block the nuclear entry of GFP::EGL-4, we asked if dynamic (that is, during prolonged odor exposure) changes in cGMP levels are able to alter the localization of EGL-4 in the AWC. To test this, we treated animals with the PDE inhibitor 3-isobutyl-1-methylxanthine (IBMX) at the time of odor exposure. This treatment is predicted to increase cGMP levels. We found that populations exposed to odor and IBMX for 80 minutes displayed higher numbers of animals with cytosolic GFP::EGL-4 when compared to populations that had been exposed to odor alone for 80 minutes. Thus, IBMX seemed to counteract odor exposure. This led us to postulate that odor decreases cGMP levels and that this decrease is required for nuclear translocation of EGL-4. Consistent with this hypothesis, expression of the phosphodiesterase PDE-3 [26] in odor naive adults increased the number of animals that exhibited nuclear GFP::EGL-4. These data suggest that decreases in cGMP levels may promote the nuclear entry of EGL-4.

We had previously shown that morphologically intact AWC cilia, as determined by fluorescent microscopic examination of the endings of the AWC neurons, are required for cytoplasmic localization of EGL-4 in an odor-naive animal [24]. In general, we define wildtype or intact cilia morphology as a fan shaped sensory ending of the AWC neuron and the absence of ectopic protrusions at the end of the dendrite. To determine whether the morphology or the function of the cilia is the key factor regulating EGL-4 localization as a function of prolonged odor exposure, we examined mutants we found in a forward genetic screen that display a constitutively nuclear EGL-4 phenotype. We found that lesions in genes required for cilia biogenesis led to cilia morphology defects, (the absence of a fan shape at the sensory ending and the presence of ectopic protrusions) and constitutive odor-independent accumulation of EGL-4 within the AWC nucleus. Chemical chelation of calcium in the adult animals had a similar effect. However, we show here using phosphodiesterase mutants that elevating cGMP levels in cilia-disrupted animals was sufficient to allow for appropriate cytoplasmic localization of EGL-4. This suggests that proper cilia morphology is required for maintaining basal cGMP levels, and that these cGMP levels even in the context of cilia defects are sufficient to allow EGL-4 to reside in the AWC cytoplasm.

cGMP signaling is an ancient and highly conserved feature of ciliary signaling. It has been noted that many eukaryotes that have lost cilia during evolution also lack cGMP signaling machinery [27]. Furthermore, the PKG EGL-4 represents a highly conserved molecular switch that shapes foraging behavior across multiple organisms [28], [29], [30], [31]. Thus, understanding the molecular mechanisms that represent the intersection between cilia and PKG activity has important implications in the sensory biology of many eukaryotes. In this work we provide evidence that the wildtype morphology of the sensory cilia may be required for a level of cGMP that keeps EGL-4 in the cytoplasm of naive AWCs. Likewise, the cilia morphology may also be required to produce odor-induced fluctuations in cGMP levels that are needed to send EGL-4 into the nucleus of the AWC.

## Results

### Changes in cGMP levels direct the nuclear entry of GFP::EGL-4 in AWC

The AWC neurons enable *C. elegans* to sense various volatile attractive odorants [6], [7]. After prolonged exposure to one of these odors, the protein kinase G EGL-4 will translocate from the cytoplasm to the nucleus of the AWC [20], [24], [25]. This nuclear entry of EGL-4 results in animals that are behaviorally adapted. Since the AWC olfactory signal transduction pathway utilizes cGMP as a second messenger [13], [14], [15], [16], and EGL-4′s ability to bind cGMP is required for its ability to translocate into the AWC nucleus [25], we decided to ask how changes in cGMP levels affect EGL-4 nuclear localization.

**Figure 1:**
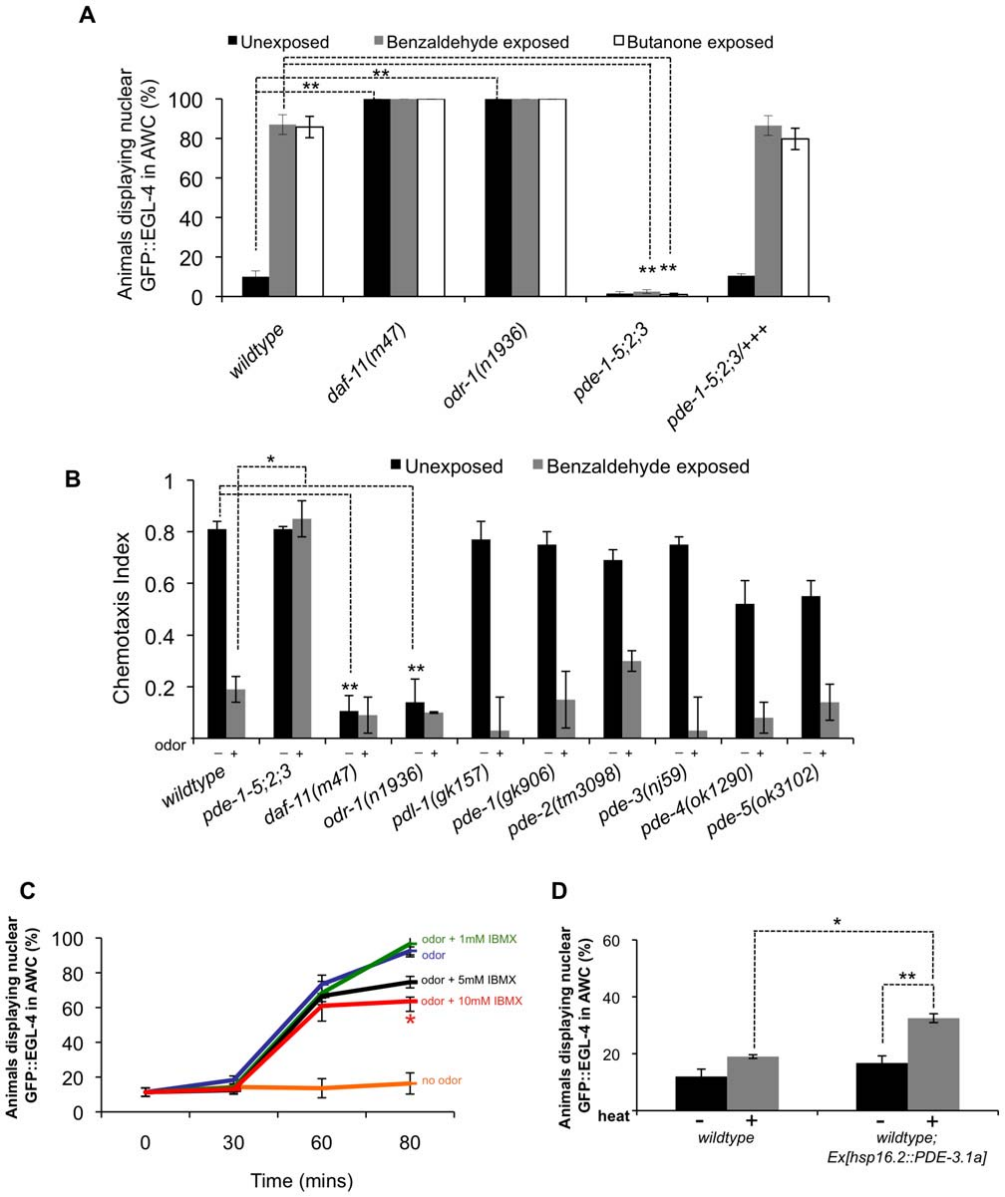
Decreases in cGMP levels direct the nuclear entry of GFP::EGL-4 in AWC and promote adaptation. (A) Percent of animals displaying nuclear GFP::EGL-4 in AWC of naive (black bars), benzaldehyde-exposed (gray bars) and butanone-exposed (white bars) treatments. **Indicates *p*≤0.005 significant differences between nuclear GFP::EGL-4 values of wildtype unadapted animals and *odr-1* or *daf-11* mutant unadapted animals, and also significant differences between wildtype adapted and *pde* quadruple mutant adapted values. (B) Chemotaxis response of PDE mutants and the guanylyl cyclase mutants *daf-11* and *odr-1* to the AWC sensed odor benzaldehyde. “−” indicates unexposed animals and “+” indicates exposed animals. **Indicates *p*≤0.005 significant differences between chemotaxis index (CI) values between wildtype unexposed animals and mutant unexposed animals. *Indicates *p*≤0.05 significant differences between wildtype odor-exposed CI values and mutant odor-exposed CI values. (C) Populations of GFP::EGL-4 (*pyIs500*) expressing animals were exposed to the odor benzaldehyde with or without the PDE inhibitor 3-isobutyl-1-methylxanthine (IBMX). Populations exposed to 10 mM concentrations of IBMX displayed a reduced number of animals exhibiting nuclear GFP::EGL-4 in AWC at 80 minutes post exposure to benzaldehyde and IBMX. *Indicates *p*≤0.05 significant differences between odor treated animals with IBMX versus odor treated animals without IBMX. (D) Expression of the cGMP phosphodiesterase PDE-3 under a heat-inducible promoter causes some increase in the number of animals displaying nuclear GFP::EGL-4. *Indicates *p*≤0.05 significant differences between wildtype and transgenic animals after heat induction; **Indicates *p*≤0.005 significant differences between transgenic animals with and without heat induction. “−” indicates no heat induction and “+” indicates after heat induction. *P* values calculated using the Student's *t*-test. Error bars represent the S.E.M.

Multiple guanylyl cyclases are expressed in the AWC [32] and two have been shown to be required for AWC mediated chemotaxis and to localize to the cilia of AWC: these are DAF-11 and ODR-1 [13], [14]. Previously, we showed that EGL-4 is constitutively nuclear in an *odr-1* mutant genetic background [25]. We examined the localization of GFP::EGL-4 in *daf-11* and *odr-1* mutants. Our laboratory has shown previously that the transgene GFP::EGL-4 is a fully functional molecule that can rescue adaptation defects in *egl-4* null mutant animals [24], [25]. We found that GFP::EGL-4 is constitutively nuclear in these guanylyl cyclase mutant animals (Figure 1A: naive wildtype = 10% nuclear, naive *odr-1(n1936)* = 100% nuclear (as seen in [24]), naive *daf-11(m47)* = 100%; *p*<0.0005 for wildtype naive animals versus *odr-1* and *daf-11* naive mutant animals). One explanation of these results is that reducing cGMP levels may promote the nuclear entry of GFP::EGL-4.

To test this in another way we examined the localization of GFP::EGL-4 in PDE mutants. There are four known cGMP PDEs in *C. elegans* [26]: PDE-1; PDE-2; PDE-3; and PDE-5. In the quadruple mutant *pde-1 pde-5; pde-2; pde-3* we found that GFP::EGL-4 is constitutively cytoplasmic. In wildtype animals after prolonged benzaldehyde exposure 87% of animals display nuclear GFP::EGL-4 and after prolonged butanone exposure 85% of animals display nuclear GFP::EGL-4. In the PDE quadruple mutant, however, after prolonged benzaldehyde exposure only 2.5% of animals display nuclear GFP::EGL-4 and after prolonged butanone exposure only 1% of animals display nuclear GFP::EGL-4 (Figure 1A: *p* = 0.0006 for wildtype benzaldehyde exposure versus PDE quadruple mutant benzaldehyde exposure, *p* = 0.0005 for wildtype butanone exposure versus PDE quadruple mutant after prolonged butanone exposure). This further suggests that decreases in cGMP levels may direct the nuclear entry of GFP::EGL-4 and increases in cGMP may block the nuclear entry of GFP::EGL-4.

Moreover, at the behavioral level, the PDE quadruple mutant is defective in its ability to adapt to AWC sensed odors (Figure 1B and Figure S1). We observed partial rescue of this behavioral defect by replacing the *pde* genes in the PDE quadruple mutant (Figure S2). To perform rescue we PCR amplified gDNA amplicons containing ∼1.5 kb upstream of the start site and ∼1 kb downstream of the stop codon of the *pde-1*, *pde-2*, and *pde-5* genes and using a fosmid containing part of the *pde-3* locus we made transgenic animals expressing these DNA fragments. As we only observed partial rescue of the adaptation behavior defect with these transgenic animals, we have not ruled the potential contribution of other background mutations that may contribute to the defect. Alternatively, there may be a precise balance required between the *pde* gene products that may not have been achieved in our attempted rescue perhaps due to incorrect levels or insufficient cis-regulatory elements. Interestingly chemotaxis behavior is intact in the PDE quadruple mutant. Wildtype animals display a chemotaxis index (CI) of 0.19 after prolonged benzaldehyde exposure. However, the PDE quadruple mutant displayed a CI of 0.85 after prolonged benzaldehyde exposure (*p* = 0.013 for wildtype versus PDE quadruple mutant after benzaldehyde exposure). Thus, chronically low cGMP (in guanylyl cyclase mutants) or high cGMP (in phosphodiesterase mutants) results in either constitutively nuclear or cytoplasmic EGL-4 respectively. Also, we did not observe adaptation defects in any of the *pde* single mutants we examined (Figure 1B).

To investigate if dynamic (that is, at the time of odor exposure) changes in cGMP levels are required to trigger the nuclear entry of EGL-4, we blocked cGMP reduction acutely by treating EGL-4::GFP expressing animals with the PDE inhibitor 3-isobutyl-1-methylxanthine (IBMX: a non-selective inhibitor of both cAMP and cGMP PDEs) during the period of prolonged odor exposure that induces nuclear translocation of EGL-4 and adaptation. In the negative control treatment, 92% of animals that were incubated with the odor benzaldehyde for 80 minutes without IBMX displayed nuclear GFP::EGL-4 (Figure 1C, blue line). This number was reduced to 63% by adding 10 mM IBMX to the odor. (Figure 1C: blue line versus red line, *p* = 0.015 for odor without IBMX versus odor with 10 mM IBMX). Incubating animals with 1 mM or 5 mM IBMX and odor had no statistically significant effect on the nuclear entry of GFP::EGL-4 (Figure 1C: *p* = 0.32 for 1 mM and *p* = 0.14 for 5 mM), and incubating with IBMX alone without odor had no effect on the localization of GFP::EGL-4 (data not shown). We also increased the time frame of IBMX exposure by pre-incubating animals in 10 mM IBMX for 45 minutes prior to the co-incident exposure of 10 mM IBMX with benzaldehyde for 80 minutes (Figure S3). However, we did not observe a greater reduction in the nuclear entry of GFP::EGL-4 by extending the IBMX treatment.

To investigate further if dynamic changes in cGMP levels trigger the nuclear entry of GFP::EGL-4, we took the opposite approach and asked whether acutely decreasing cGMP levels in adults might be sufficient to send EGL-4 into the nucleus of a naive animal. Thus, we overexpressed the cGMP phosphodiesterase PDE-3 under a heat-inducible promoter (*hsp16.2::pde-3.1a*) in odor-naive animals. Inducing the overexpression of *pde-3* in wildtype, odor naive adult animals resulted in modest but significantly higher numbers of animals displaying nuclear GFP::EGL-4 (Figure 1D). 32.5% of transgenic animals expressing the *hsp16.2::pde-3.1a* transgene exhibit nuclear GFP::EGL-4 after heat induction versus 19% of non-transgenic wildtype animals (*p* = 0.04). Taken together, these data argue that reducing or increasing cGMP levels can dynamically modulate the nuclear entry of GFP::EGL-4.

### A forward genetic screen for constitutively nuclear GFP::EGL-4 mutant animals

**Figure 2:**
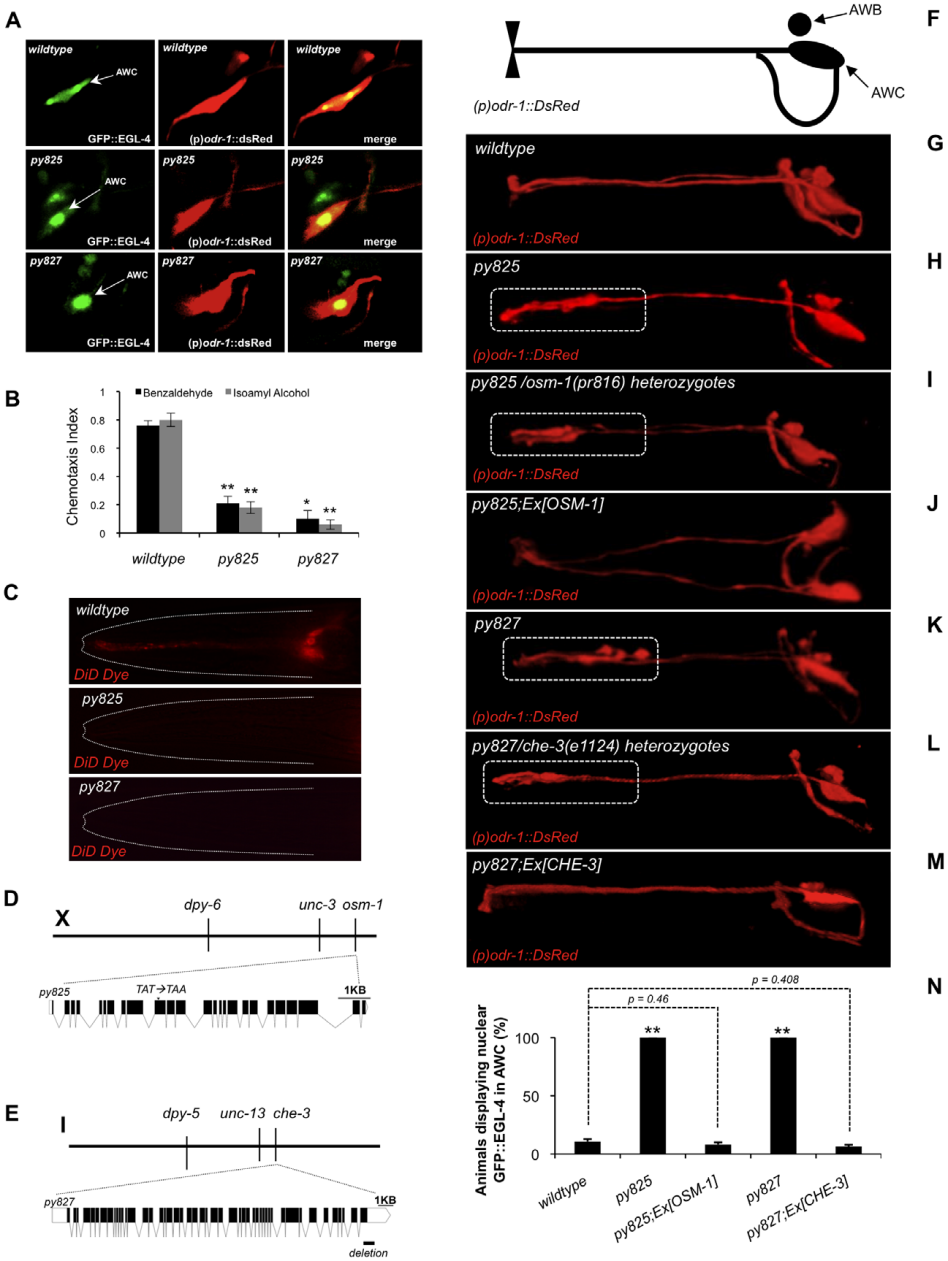
Forward genetic screen revealed that cilia defective mutants show constitutively nuclear EGL-4. (A) In wildtype naive animals GFP::EGL-4 is diffuse throughout the entire cytosol of AWC, in the mutant *py825*, GFP::EGL-4 is constitutively accumulated in the nucleus of AWC and GFP::EGL-4 is seen constitutively accumulated in the nucleus of AWC in the mutant *py827*. The constitutively nuclear GFP::EGL-4 mutants *py825* and *py827* are chemotaxis and dye filling defective. (B) Chemotaxis responses of wildtype animals and the mutants, *py825* and *py827* to the AWC sensed odors benzaldehyde (black bars) and isoamyl alcohol (gray bars). **Indicates *p*≤0.005, and *indicates *p*≤0.05 significant differences between mutants and wildtype animals for each odor. (C) Dye filling with the lipophillic dye DiD in wildtype, *py825*, and *py827* animals. The amphid neurons of the mutants *py825* and *py827* fail to dye fill. (D) *py825* encodes OSM-1, an ortholog of the intraflagellar transport (IFT) complex B component, IFT172. Three factor mapping with *dpy-6 unc-3* placed *py825* close to *unc-3*. The molecular lesion of *py825* is indicated by an arrowhead. (E) *py827* encodes CHE-3, the isoform 1b of the dynein heavy chain (DHC). Gene structure of *che-3* and molecular lesion of *py827*. The molecular lesion of *py827* is indicated in the gene structure by a black horizontal line. Three factor mapping with *dpy-5 unc-13* placed *py825* near *unc-13*. Exon intron graphic generated using the web application at: http://wormweb.org/exonintron. (F) Cartoon illustrating the features of the (p)*odr-1*::DsRed expression pattern in the subsequent fluorescent confocal images. (G) Normal AWC cell and cilia morphology in wildtype animals. (H) AWC cilia defects displayed by *py825*. (I) AWC cilia defects observed in *py825/osm-1(pr816)* trans heterozygotes. (J) Normal cilia morphology observed in *py825* transgenic animals expressing a rescuing array of OSM-1. (K) AWC cilia defect displayed by *py827*. (L) AWC cilia defects observed in *py827/che-3(e1124)* trans heterozygotes. (M) Normal cilia morphology observed in *py827* transgenic animals expressing a rescuing array of CHE-3. (N) Quantification of the constitutively nuclear GFP::EGL-4 phenotype in *py825* and *py827* mutant animals. In a wildtype population very few animals display nuclear GFP::EGL-4. The mutants *py825* and *py827* both display nuclear GFP::EGL-4 in all animals. The nuclear GFP::EGL-4 mutant phenotype of *py825* is reduced to wildtype levels in transgenic mutant animals expressing the rescuing array containing OSM-1, and similarly for *py827* the nuclear GFP::EGL-4 mutant phenotype is reduced to wildtype levels in transgenic mutant animals expressing the rescuing array containing CHE-3.**Indicates *p*≤0.005 significant differences between wildtype and mutant animals. *P* values calculated using the Students *t*-test. Error bars represent the S.E.M. For all images anterior is to the left, and white dotted lines indicate outline of animal's head region in (C) and white dotted rectangles indicate cilia structural defects in (H), (I), (K) and (L).

To identify determinants of the nuclear translocation of EGL-4 in AWC we conducted a forward genetic screen to isolate mutants that display constitutively nuclear GFP::EGL-4. Two mutants isolated from this screen are *py825* and *py827* (Figure 2A). Interestingly, *py825* and *py827* are defective in their ability to respond to AWC sensed odors (Figure 2B: wildtype benzaldehyde chemotaxis index [CI] = 0.76, *py825* benzaldehyde CI = 0.21 [*p* = 0.008 compared to wildtype], *py827* benzaldehyde CI = 0.102 [*p* = 0.01 compared to wildtype], wildtype isoamyl alcohol CI = 0.8, *py825* isoamyl alcohol CI = 0.18 [*p* = 0.003 compared to wildtype], and *py827* isoamyl alcohol CI = 0.06 [*p* = 0.003 compared to wildtype]), and are also defective in adaptation responses (Figure S4). They are also defective in their ability to uptake the lipophillic dye DiD (Figure 2C). This dye will fill sensory neurons that have ciliated endings exposed to the external environment (Figure 2C, top panel ‘wildtype’), so this indicated that the cilia have been disrupted. The mutant *py825* was mapped to the right arm of LGX close to *unc-3* (Figure 2D). Complementation tests were performed with mutants in that region and consequently we found that *py825* was allelic to *osm-1*. The AWC cilia defect and constitutively nuclear GFP::EGL-4 mutant phenotype could be rescued by introducing the array (p)*osm-1*::OSM-1 into the genetic background of *py825* (Figures 2J and 2N). GFP::EGL-4 is nuclear in 100% of *py825* animals. In *py825* transgenic animals expressing the rescuing array (p)*osm-1*::OSM-1, the nuclear GFP::EGL-4 phenotype is reduced to 8%. This is statistically indistinguishable from wildtype animals (Figure 2N, *p* = 0.46). OSM-1 is an ortholog of the intraflagellar transport (IFT) complex B component, IFT172 [33], [34]. In sequencing the *osm-1* locus, we found that the *py825* lesion is caused by a premature ochre stop codon in the tenth exon (Figure 2D).

The mutant *py827* was mapped near the center of LGI, close to *unc-13* (Figure 2E). Through complementation testing we then found that *py827* was allelic to *che-3*, and both the AWC cilia defect and the constitutively nuclear GFP::EGL-4 mutant phenotype could be rescued by introducing the array (p)*che-3*::CHE-3 into the genetic background of *py827* (Figures 2M and 2N). 100% of *py827* mutant animals displayed nuclear GFP::EGL-4. This was reduced to 6.33% in transgenic *py827* animals expressing the rescuing array (p)*che-3*::CHE-3. This is statistically indistinguishable from wildtype animals (Figure 2N, *p* = 0.408). The gene *che-3* encodes the isoform 1b of the dynein heavy chain (DHC) [33], [34], [35]. Sequencing of the *che-3* locus in *py827* revealed that the lesion is defined by a 397 bp deletion that removes the final 54 bps of the final exon of the *che-3* gene (Figure 2E and Figure S5). Since the most prominent defects of mutants that exhibit 100% nuclear EGL-4 in the naive animal are cilia defects, this led us to propose that proper cilia morphology is important for the cytosolic distribution of EGL-4 in the naive animal. This corroborates our previous study [24] in which we noted that the severity of a strain's cilia defects correlated well with the percent of the population that exhibits constitutively nuclear EGL-4.

### The localization of GFP::EGL-4 does not alter AWC cilia morphology

**Figure 3:**
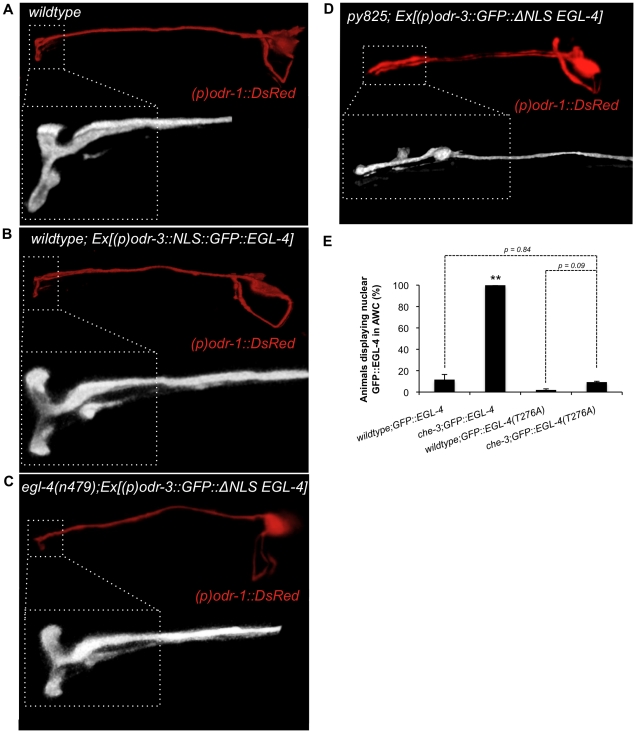
Subcellular localization of GFP::EGL-4 in AWC does not affect cilia morphology. (A) Fluorescent confocal image of the AWC neuron in a wildtype animal. Inset is a magnified view of the AWC cilia. (B) Fluorescent confocal image of wildtype animal expressing a constitutively nuclear form of GFP::EGL-4. The animal displays normal AWC cilia. Inset is a magnified view of the AWC cilia. (C) An *egl-4(n479)* mutant animal expressing constitutively cytosolic GFP::EGL-4 displays normal AWC cilia. Inset is a magnified view of the AWC cilia. (D) Though the *py825* mutant strain has constitutively nuclear EGL-4, expressing a cytosolic version of GFP::EGL-4 does not rescue the ciliopathy of *py825* mutant animals. (E) Quantification of the localization of GFP::EGL-4 in AWC. In a wildtype population very few animals display nuclear GFP::EGL-4. In the mutant *che-3(e1124),* GFP::EGL-4 is in the nucleus of all animals. Mutating a key residue in the cGMP binding site of EGL-4 prevents the nuclear entry of GFP::EGL-4 in *che-3(e1124)* mutant animals.** indicates statistical significance at *p*<0.005 between wildtype and *che-3* mutant animals For all images anterior is to the left, and white dotted lines indicate outline of animal's head region. *P* values calculated using the Student's *t*-test. Error bars represent the S.E.M.

Neuronal plasticity is often accompanied by structural changes [36], [37], [38]. This structural plasticity involves transcriptional changes that are regulated by multiple kinases. These include: Ca^2+^/calmodulin kinase CAMKII [39]; cyclin dependent kinase CDK5 [40]; tyrosine receptor kinase TrKB [41]; and LIM-kinase (LIMK) [42], as well as many others. To test the hypothesis that the localization of the PKG EGL-4 causes defects in the morphology of the AWC cilia we asked whether changes in cilia morphology occur as a result of the subcellular localization of EGL-4. To examine this, we expressed a constitutively nuclear form of EGL-4 in AWC. This transgene of EGL-4 contains a second nuclear localization sequence (NLS) and is thus constitutively nuclear [25]. In animals containing this transgene of EGL-4 the cilia of the AWC were normal (Figure 3B and Table S1). We also examined whether overexpressing EGL-4 in the cytoplasm of AWC could alter the morphology of the cilia in AWC. To this end, we expressed a transgene of EGL-4 in which the NLS had been deleted, causing EGL-4 to be constitutively cytoplasmic [25]. *egl-4* mutant (*n479* - null) animals expressing this transgene exhibited normal cilia (Figure 3C and Table S1). Thus, the localization of EGL-4 does not affect cilia morphology.

Though the above experiments did not identify a role for EGL-4 localization in directing cilia morphology in the wildtype animal, we next wanted to determine whether it might play a role in cilia morphology that would be uncovered in the context of the IFT complex B mutant background. Thus we asked whether we could ameliorate the AWC cilia defect of *osm-1(py825)* mutant animals by overexpressing the constitutively cytoplasmic GFP::EGL-4. In this scenario we observed no rescue of the ciliopathy (Figure 3D and Table S1). Taken together, these data suggest that the morphology of the AWC cilia is not affected by the localization of EGL-4 but rather EGL-4′s localization is affected by the morphology of the AWC cilia.

We wanted to understand whether the nuclear localization of EGL-4 in mutants with defects in cilia morphology is dependent upon its ability to bind cGMP. We examined a mutation (T276A) within the low affinity binding site of EGL-4. Our decision to examine a mutation affecting the low affinity cGMP binding site was guided by previous studies into cGMP binding within PKGs. These studies have shown that there are two cGMP bindings sites (low affinity and high affinity) and they act allosterically to activate the kinase domain with a Hill coefficient of 1.6 [43]. Initially, cGMP saturates the high affinity site. When the high affinity cGMP binding site is saturated the kinase becomes 50% active and then the low affinity cGMP binding site is primed for cGMP binding. When cGMP saturates the low affinity site the enzyme becomes fully active. Thus, we inferred from biochemical studies of PKGs that mutating either cGMP binding site would dramatically decrease the ability of EGL-4 to perceive cGMP changes. In fact, we previously demonstrated that mutation of key residues within either cGMP binding site (low affinity or high affinity) of EGL-4 resulted in a complete block in EGL-4′s ability to translocate to the nucleus after prolonged odor exposure [25]. Thus, we mutated the low affinity cGMP binding site within EGL-4, and examined its localization in the *che-3* mutant background. Indeed, we found that mutating a key residue in one of the cGMP binding sites (T276A) suppressed the constitutively nuclear GFP::EGL-4 phenotype of *che-3* mutant animals (Figure 3E). *che-3(e1124)* mutant animals display nuclear GFP::EGL-4 in 100% of cases. In *che-3(e1124)* transgenic animals expressing GFP::EGL-4(T276A), in which the cGMP binding site is mutated, the nuclear GFP::EGL-4 phenotype is reduced to 4%. This is statistically indistinguishable from the percent of naive wildtype animals displaying nuclear GFP::EGL-4 (*p* = 0.84), and also indistinguishable from the number of naive wildtype transgenic animals expressing the transgene GFP::EGL-4(T276A) (*p* = 0.09). Importantly, the cilia defects of *che-3(e1124)* were not altered by expression of this constitutively cytosolic form of EGL-4 (data not shown). These data suggest that the native cGMP binding sites within EGL-4 are required for nuclear entry of EGL-4, even in a cilia biosynthesis mutant background such as *che-3(e1124),* which normally exhibits a constitutively nuclear EGL-4 phenotype. Mutation of one of the cGMP binding sites within EGL-4 may disrupt proper cGMP occupancy that could normally direct autophosphorylation or heterophosphorylation events important for the nuclear entry of EGL-4. Alternatively, by interfering with the ability of EGL-4 to bind cGMP, we may have altered the conformation of EGL-4 in a way that inhibits its ability to translocate to the nucleus.

### High cGMP levels result in cytosolic EGL-4 even when cilia morphology is defective

**Figure 4:**
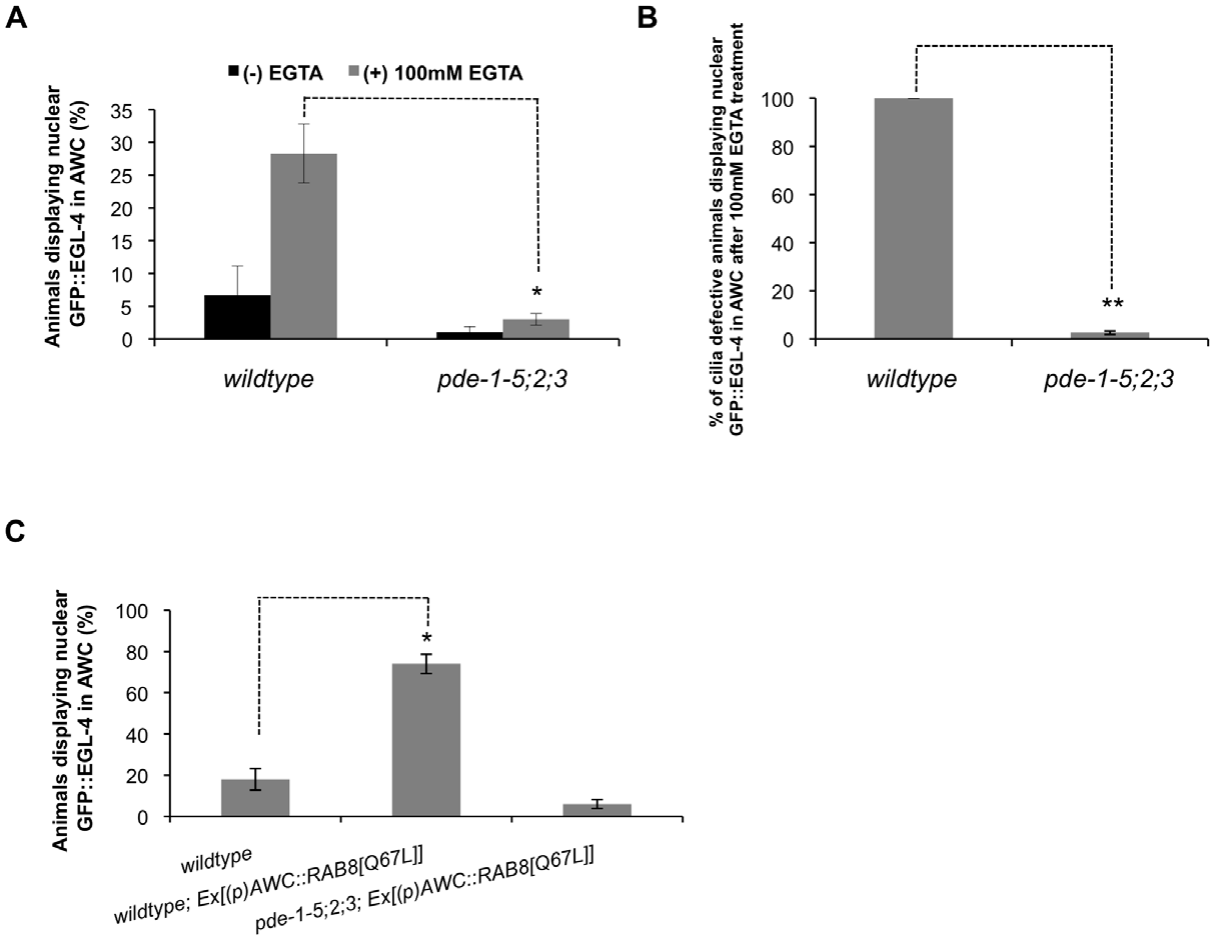
cGMP levels are more important for regulating EGL-4 subcellular localization than is cilia morphology. (A) Populations of naive wildtype animals that are soaked in high concentrations of the calcium chelating agent EGTA display significantly higher numbers of animals with nuclear GFP::EGL-4, however, *pde-1-5;-2;-3* mutant animals display significantly fewer numbers of nuclear GFP::EGL-4 animals after EGTA treatment. *Indicates *p*≤0.05 significant differences between wildtype EGTA treated animals and *pde-1-5;-2;-3* mutant EGTA treated animals. (B) The percentage of cilia defective animals displaying nuclear GFP::EGL-4 after 100 mM EGTA treatment was compared between wildtype and *pde-1-5;2;3* mutant animals. For wildtype animals 100% of cilia defective animals exhibited nuclear GFP::EGL-4 and for *pde-1-5;2;3* mutant animals 2.6% of cilia defective animals displayed nuclear GFP::EGL-4. (C) Transgenic animals expressing the constitutively active form of RAB-8 in AWC displayed defects in cilia morphology. We examined the localization of GFP::EGL-4 in wildtype and PDE quadruple transgenic animals expressing RAB-8[Q67L] that exhibit defects in cilia morphology and compared to the percentage of nuclear localized GFP::EGL-4 in wildtype animals. 18% of wildtype animals display nuclear GFP::EGL-4, 74% of cilia defective wildtype animals expressing RAB-8[Q67L] display nuclear GFP::EGL-4, and 6% of PDE quadruple mutants expressing RAB-8[Q67L] in AWC display nuclear GFP::EGL-4. Between 30 and 60 cilia defective animals were counted for each trial and the means of three separate trials conducted on separate days was graphed. **Indicates *p*≤0.0005 significant differences between wildtype EGTA treated cilia defective animals and *pde-1-5;-2;-3* mutant EGTA treated cilia defective animals. *Indicates *p*≤0.005 significant differences between wildtype animals expressing RAB-8[Q67L] in AWC compared to *pde-1-5;-2;-3* mutant animals expressing RAB-8[Q67L] in AWC. *P* values calculated using the Students t-test. Error bars represent the S.E.M.

Our data so far describes a model whereby the AWC cilia facilitate changes in cGMP levels, and these changes are interpreted by EGL-4 to determine its proper localization. Next, we tested if GFP::EGL-4 is cytosolic in the PDE quadruple mutant background even when cilia morphology is perturbed. Calcium has been shown to be critical in maintaining cilia morphology in *C. elegans* [44], and we found that by soaking animals in high concentrations of the calcium-chelating agent ethylene glycol tetraacetic acid (EGTA), we could both induce perturbations of cilia morphology in 22% of treated animals (Figure S6), and increase the percent of animals with nuclear GFP::EGL-4 (Figure 4A). We define perturbation of cilia as an absence of the cilia fan shape often accompanied by ectopic protrusions at the distal tip. However, after soaking the PDE quadruple mutant animals in high concentrations of EGTA we did not observe a comparable increase in animals displaying nuclear GFP::EGL-4 (Figure 4A: wildtype animals after EGTA treatment = 28.3%, PDE quadruple mutant animals after EGTA treatment = 3%, *p* = 0.01). They did, however, show evidence of cilia defects that were comparable to the EGTA-treated wildtype animals (data not shown). In fact, even without EGTA treatment a subset of PDE quadruple mutants exhibit a decreased AWC cilia fan shape size, and these animals still display cytosolic GFP::EGL-4. We also compared the percentage of cilia defective animals exhibiting nuclear GFP::EGL-4 after 100 mM EGTA treatment (Figure 4B) between populations of wildtype animals and *pde-1-5;2;3* quadruple mutant animals. In the wildtype population, we observed that 100% of animals that had cilia defects also had nuclear GFP::EGL-4, while in the *pde-1-5;2;3* quadruple mutant population, only 2.6% of cilia defective animals displayed nuclear GFP::EGL-4 (Figure 4B: *p*<0.0005).

Next we used a genetic manipulation to examine the epistatic relationship between elevated cGMP and cilia morphology in AWC with respect to the localization of GFP::EGL-4. Previously it has been shown that over-expression of a constitutively active form of the small GTPase RAB-8 resulted in cilia defects in the AWB neurons in *C. elegans* [44]. We expressed the constitutively active form of RAB-8[Q67L] under an AWC exclusive promoter in wildtype animals expressing GFP::EGL-4 and PDE quadruple mutants expressing GFP::EGL-4. In most transgenic aimals expressing RAB-8[Q67L] we did not observe the fan-shaped cilia of AWC and often observed ectopic protrusions at the distal tip (Figure S7). Furthermore, in the animals displaying perturbed cilia morphology we observed nuclear GFP::EGL-4 in 74% of these animals versus 6% in the PDE quadruple mutant background (Figure 4C: *p* = 0.001 between wildtype animals expressing RAB-8[Q67L] and PDE quadruple mutants expressing RAB-8[Q67L]). Taken together, this suggests that elevated cGMP levels are epistatic to changes in cilia morphology in relation to the localization of EGL-4.

## Discussion

The elaborate structure of the AWC cilia is exquisitely formed for function. The petal-shaped cilia provide a large membranous area within which sensory and transduction proteins are localized. This structure both concentrates and compartmentalizes sensory signal transduction components. In concert with intracellular signaling proteins, the factors within the AWC cilia provide a dynamic response range as well as functional plasticity to the AWC neuron.

The protein kinase G EGL-4, which is found throughout the AWC cytoplasm including the cilia, is a central regulator of plasticity in the AWC neuron [20], [23], [25]. Sustained stimulation of the AWC neuron triggers nuclear entry of EGL-4; this nuclear entry is both necessary and sufficient to dampen sensory responses of the AWC neuron [25]. The molecules and proteins that instruct this dynamic event are not fully known. The aim of our current study was to shed light on the proteins and molecules that comprise the intersection between cilia and EGL-4 activity in the AWC neuron. Using the localization of EGL-4 as a read-out, we performed both a candidate screen and a forward genetic screen to search for genes that are required for proper localization of EGL-4 in the AWC neuron. From these screens, we found that cGMP levels whose dynamic changes likely require intact cilia morphology are important for proper localization of EGL-4.

Non-motile cilia, like that of the AWC, are developed and maintained by intraflagellar transport (IFT). IFT employs the anterograde motor, Kinesin, to traffic ciliary cargo to the growing end of the ciliary structure, and uses the retrograde motor, cytoplasmic Dynein 1b, to traffic cargo back to the base of the cilium [45], [46], [47]. These motors are associated with large multi-protein assemblies called IFT complex A and IFT complex B. Another assembly, the BBSome, stabilizes trafficking of the motors/IFT particles A, B/cargo complex along the ciliary axoneme [48], [49], [50]. Several proteins that comprise part of the sensory machinery of AWC have been shown to localize to the cilia. These include the guanylyl cyclases DAF-11 and ODR-1 [13], [14]. Guanylyl cyclases catalyze the reaction of guanosine triphosphate (GTP) to 3′,5′-cyclic guanosine monophosphate (cGMP) and pyrophosphate. The cGMP product serves as a second messenger capable of activating numerous targets including protein kinases and ion channels. To understand how cGMP changes determine the localization of GFP::EGL-4, we examined the localization of EGL-4 in mutants of DAF-11 and ODR-1. We found that, in these mutants, a functional GFP tagged EGL-4 is constitutively localized to the AWC nucleus. This is consistent with what we had previously observed in the *odr-1(null)* mutants [24]. This suggested to us that reducing cGMP levels in AWC might direct the nuclear entry of GFP::EGL-4. If the levels of cGMP were indeed required to drop to promote EGL-4 nuclear entry, we would expect that EGL-4 nuclear entry would be blocked in animals in which cGMP levels cannot decrease. To test this, we asked whether EGL-4 could enter the nucleus in mutants that lacked cGMP phosphodiesterases. cGMP phosphodiesterases break the phosphodiester bond within cGMP; there are four predicted cGMP phosphodiesterases in the *C. elegans* genome [26]. We examined the localization of GFP::EGL-4 in a quadruple mutant of all four cGMP phosphodiesterases, and found that GFP::EGL-4 was constitutively cytosolic in this mutant background. This further suggested that decreasing cGMP levels in the AWC may promote the nuclear entry of EGL-4 and, inversely, increasing cGMP levels in AWC blocks the nuclear entry of GFP::EGL-4.

Interestingly, in our previous studies, over expression of the guanylyl cyclase ODR-1 did not have the same effect as loss of PDE expression [24]. In these previous studies, we found that over expressing the guanylyl cyclase ODR-1 blocked adaptation, which is similar to the loss of the PDEs. However, over expression of ODR-1 did not block nuclear entry of EGL-4. Thus, the levels of cGMP may not be raised sufficiently by over expression of ODR-1 to block nuclear entry but adaptation may require additional events downstream of EGL-4 nuclear entry that over expression of ODR-1 blocks. A better understanding of this discrepancy between over expressing ODR- 1 and loss of the PDEs may be facilitated by cGMP imaging studies.

We also demonstrated that dynamic changes in cGMP levels in adults through over expression of the phosphodiesterase PDE-3 under a heat-inducible promoter or pharmacological treatment with IBMX both modestly affected the nuclear entry of GFP::EGL-4 in AWC. The discrepancy between the marked extent of our ability to alter nuclear EGL-4 levels by genetic means (genetic loss of PDEs), which resulted in a high percentage of cytoplasmic EGL-4 animals in the presence of odor, versus the modest block in nuclear translocation by acute inactivation of the PDEs by IBMX might indicate either that the pharmacological manipulations are less effective or that the PDEs are required for additional developmental events that allow EGL-4 to enter the AWC nucleus. Likewise, acute expression of a PDE from the heat-inducible promoter was only able to promote nuclear translocation in a small percent of the treated animals. This could indicate that the treatment did not adequately reduce cGMP levels or that additional factors, perhaps phosphorylation events downstream of Gα activation, are required to promote nuclear entry of EGL-4. Once again, cGMP-based imaging may help to distinguish between these possibilities.

Interestingly, chemotaxis was not affected by loss of the PDEs in contrast to adaptation, which was completely abolished. This indicates that dynamic fluxes of cGMP via PDE may be less important for acute odor recognition than adaptation. Although, there may be alternate factors or pathways required to modulate cGMP levels to allow for acute odor recognition. In a recent study of the photoreceptor cell ASJ in *C. elegans*, Liu *et al*. [26] demonstrated that phototransduction in ASJ is mediated by cGMP signaling through guanylyl cyclases and CNG channels. Interestingly, photocurrents were observed in *pde-1 pde-5; pde-2; pde-3* quadruple mutants but exhibited very slow recovery, suggesting that cGMP levels may be modulated independently of PDE activity. Perhaps AWC exhibits a similar cascade, whereby acute odor recognition is executed independent of PDE activity, and more long-term events such as olfactory adaptation are defined by PDE modulation of cGMP levels.

Though we have implicated fluctuations in cGMP as being important for directing EGL-4 localization and initiating olfactory adaptation, to develop a complete picture of how cGMP dynamics shape AWC mediated behaviors, we will need to develop temporal and spatial indicators of cGMP levels. Recently, several groups have reported their data on a cGMP-sensitive GFP molecule [51], [52]. Developing this tool for use in *C. elegans* may provide information on the temporal activity of cGMP in AWC, during both acute odor recognition and sustained odor stimulation.

## Materials and Methods

### Strains and maintenance

Bristol N2, *osm-1(py825), che-3(py827), dpy-6(e14) unc-3(e151), dpy-5(e61) unc-13(e1091), osm-1(pr816), che-3(e1124), py825; Ex[OSM-1], py827; Ex[CHE-3], egl-4(n479),* N2; *pyIs500[(p)odr-3*::GFP::EGL-4; *(p)odr-1*::DsRed; *(p)ofm-1*::GFP], N2; Ex[*(p)odr-3*::NLS::GFP::EGL-4], *py825*; Ex[*(p)odr-3*::GFP::ΔNLS EGL-4], *egl-4(n479)*; Ex[*(p)odr-3*::GFP::ΔNLS EGL-4], N2; Ex[*(p)odr-3*::GFP::EGL-4 *(T276A)], che-3(e1124)*; Ex[*(p)odr-3*::GFP::EGL-4 *(T276A)], daf-11(m47), daf-11(m47); pyIs500, odr-1(n1936), odr-1(n1936); pyIs500, pde-1(nj57) pde-5(nj49); pde-2(tm3098); pde-3(nj59)*; Ex[*(p)odr-3*::GFP::EGL-4;*(p)odr-1*::DsRed; *(p)ofm-1*::GFP], *pde-1(nj57) pde-5(nj49); pde-2(tm3098); pde-3(nj59), pde-1(nj57) pde-5(nj49); pde-2(tm3098); pde-3(nj59)*; Ex[*(p)ceh-36*::RAB8[Q67L], N2; Ex[*(p)ceh-36*::RAB8[Q67L], *unc-5(e53)*IV; *dpy-11(e224)*V; *lon-2(e678)*X, *dpy-5(e61)*I; *bli-2(e768)*II; *unc-32(e189)*III, *pyIs500*; Ex[*elt-2*::GFP, *hsp16.2::pde-3.1a]*. All strains were maintained on NGM plates that were seeded with *E. coli* strain OP50 according to standard protocol [53].

### Constitutively nuclear GFP::EGL-4 screen

L4 hermaphrodite *pyIs500* animals were mutagenized using 50 mM of EMS as described previously [53]. 4000 haploid genomes were screened as an F2 microscope screen. Single F1 progeny were cloned onto individual OP50 seeded NGM plates and F2 progeny were subsequently examined under 40× magnification for the constitutively nuclear GFP::EGL-4 phenotype. Mutant animals were rescued from microscope slides and their progeny examined for transmission of the mutant phenotype.

### Mapping of *py825* and *py827*

The *py825* mutant was assigned to LGX by the following cross: *py825* hermaphrodites were crossed with wildtype males and F1 male cross progeny were examined for the constitutively nuclear GFP::EGL-4 phenotype - all male cross progeny were mutant for the GFP::EGL-4 localization phenotype suggesting that *py825* is X-linked. Three factor mapping with *unc-3 dpy-6* animals mapped *py825* very close to *unc-3* (21/21 recombinant *unc-3* non *dpy-6* animals were wildtype for the GFP::EGL-4 phenotype and 9/9 *dpy-6* non *unc-3* recombinants displayed the constitutively nuclear GFP::EGL-4 phenotype). Complementation tests were performed with mutant animals harboring lesions in the chromosomal region of *unc-3* and from this we found that *py825* and *osm-1(pr816)* are allelic. The *py827* mutant phenotype was assigned to LGI using the mapping strains MT464 and MT465 (3/15 MT464 *unc-5* IV non *dpy* V non *lon* X recombinant animals were wildtype for the GFP::EGL-4 phenotype, and 3/15 MT464 *lon-2* non *dpy* non *unc* recombinants were wildtype for the GFP::EGL-4 phenotype, and 1/14 MT465 *dpy-5* I non *bli* II non *unc* III recombinants were mutant for the GFP::EGL-4 phenotype). Three factor mapping with *unc-13 dpy-5* animals placed *py827* very close to *unc-13* (7/7 *unc-13* non *dpy-5* recombinants were wildtype for GFP::EGL-4 localization and 4/7 *dpy-5* non *unc-13* recombinant animals were mutant for the GFP::EGL-4 phenotype). Complementation tests with animals harboring mutations in this chromosomal region were performed and it was found that *py827* and *che-3(e1124)* are allelic. AWC cilia surface area was measured for *osm-1*(*py825)* and *che-3*(*py827*) (see Table S1 and Figure S8 for details). The *osm-1(py825)* lesion is caused by a premature ochre stop codon in the tenth exon changing a tyrosine (TAT) at amino acid position 496 to a STOP (TAA). The *che-3*(*py827*) lesion is defined by a 397 bp deletion that removes the final 54 bps of the final exon of the *che-3* gene and can be genotyped using the following primer pair: jz827-F GACTCCTTCTCAACTACCAGCTAAACAATG and jz827-R CATCTGCGAGACGTACTGATAGAATACAAG.

### Dye filling assays

The lipophillic dye DiD (Invitrogen) was eluted in 2.5 ml of dimethylformamide (DMF) to obtain a stock solution of 10 mg/ml. Worms were washed from OP50 seeded plates two times in M9 solution and exposed to DiD dye (1∶1000) on a rotator for 2 hours. The worms were then washed two times in M9 and allowed to recover on an OP50 seeded plate for 1 hour. The animals were then washed off the plate two times in ddH_2_0 and examined using a TRITC filters under 40× magnification.

### Chemotaxis and adaptation assays

Chemotaxis was performed as described previously [7], [20], [23]. Briefly, four to five L4 animals were picked from plates grown at 25°C onto a 10 cm OP50 seeded plate and incubated at 25°C. Animals were washed from these plates and chemotaxis and adaptation assays were performed at room temperature. Odors were diluted as follows unless otherwise stated: 1 µl benzaldehyde (Sigma) in 200 µl EtOH, 1 µl butanone (Sigma) in 1000 µl EtOH, and 1 µl isoamyl alcohol (Sigma) in 100 µl EtOH. Odor adaptations were performed as described elsewhere [23], [24]. Adaptation mixes were prepared by diluting odors as follows; 7.5 µl benzaldehyde into 100 ml S-Basal buffer, 11 µl butanone into 100 ml S-Basal buffer and populations of animals were soaked in the diluted odor for 80 minutes during long-term exposure. For all assays between 100 and 200 animals were assayed in each assay and each assay was repeated on separate days between three and five times.

### EGL-4 nuclear translocation assays

Four to five L4 animals were picked onto a 10 cm OP50 seeded plate and incubated at 25°C. Animals were washed from these plates and translocation assays were performed by exposing mutant animals containing the GFP::EGL-4 transgene to odor (adapted) or S-Basal (unadapted) for 80 minutes and then scoring the number of worms exhibiting EGL-4 in the AWC ‘ON’ nucleus after butanone exposure or in both AWC nuclei after benzaldehyde exposure under 40× magnification. Wildtype *pyIs500* worms were included for every translocation assay as a positive control using ≥75^th^ percentile as the baseline for successful control assays and treatment assay inclusion. Between twenty and fifty animals were scored for each translocation assay and repeated on separate days three to five times.

### EGTA, IBMX treatment and heat shock protocol

For EGTA (Sigma) treatment, wildtype and *pde-1 pde-5; pde-2; pde-3* mutant animals containing the transgene GFP::EGL-4 (*pyIs500*) were incubated in 100 mM EGTA (pH 7.0) for 60 minutes and the subcellular localization of EGL-4 in AWC was examined as described. For IBMX (Sigma) treatment, GFP::EGL-4 (*pyIs500*) expressing animals were incubated with adaptation solutions containing various IBMX concentrations (1 mM, 5 mM, 10 mM). Between 50 and 80 animals were counted for each assay, and the assay was repeated in three separate trials. For the heat-induced expression of *pde-3.1a*, ∼5 L4 *pyIs500* (GFP::EGL-4) transgenic animals containing the *hsp16.2::pde-3.1a* transgene were grown at 15°C for ∼8 days, then washed 3 times in S-basal buffer and placed on an unseeded plate at 30°C for 2 hours. Then the subcellular localization of EGL-4 in AWC was examined microscopically as described. Between 30 and 50 animals were examined on each day for both the control and experimental populations and repeated in four separate trials on different days.

### Plasmid construction and transgenic strains

Construction of (p)*odr-3*::GFP::EGL-4, (p)*odr-3*::GFP::NLS::EGL-4, (p)*odr-3*::GFP::EGL-4ΔNLS and (p)*odr-3*::GFP::EGL-4(T276A) was described previously [25]. The (p)*ceh-36*::RAB8[Q67L] plasmid was generated by PCR amplifying the RAB-8[Q67L] cDNA with primers flanked with *Eco*RI and *Kpn*I sites, the amplicon was digested with *Eco*RI and *Kpn*I and inserted into a (p)*ceh-36* containing plasmid in pPD95.75. The OSM-1 rescuing array was a gift from Lesilee Rose, and the WRM0637cB09 fosmid library clone was used for the CHE-3 rescuing array. The PCR fusion *hsp16.2::pde-3.1a* was made by PCR amplifying the heat shock promoter *hsp-16.2* and *pde-3.1a* gene (from ATG start codon to ∼1 kb downstream of stop codon) from genomic DNA and fusing these products together by PCR fusion as described previously [54]. The primer pair used to amplify the heat shock promoter *hsp16.2* was: hspF gatcaagagcatttgaatcagaatatgg and hspR pde-3 fuse ttttgatttaaaaaccttggagttcatagtgagatgattatagtttgaagatttctaat. The primer pair used to amplify the *pde-3.1a* product was: pde-3F atgaactccaaggtttttaaatcaaaaaaaa and pde-3R gaaaaagggcagttttgttgtgtatgc. These products were fused using the nested primer pair: hspF nest agttttttagatgcactagaacaaagcgtg and pde-3R nest gggcagttttgttgtgtatgcaaat. Rescuing *pde* transgenes were generated by PCR amplifying gDNA fragments containing ∼1.5 kb upstream of the start site and ∼1 kb downstream of the stop codon of the *pde-1*, *pde-2*, and *pde-5* genes. In the case of *pde-3* we used the fosmid WRM0623dE11 which contains part of the *pde-3* locus. The PCR amplicons and fosmid were co-injected together at 5 ng/µl each and transgenic lines were assayed for rescue of the PDE quadruple adaptation behavior defect. The following primer pairs were used to amplify gDNA fragments from the *pde-1*, *pde-2* and *pde-5* loci: pde-2F ttatcagctgattatttcgttttagtgatagtg and pde-2R caccatgatctctaattataatcccgc; pde-1F acaaaaatgtgttcaactgatacaattttc and pde-1R acttgaactcggtgcggattccggaatc; pde-5F acgattcctacaaactacgaataccctac and pde-5R gttctttgaaccaaaaggaaggcatac.

## Supporting Information

Figure S1 **Chemotaxis response of the PDE quadruple mutant and the guanylyl cyclase mutants** ***daf-11*** **and** ***odr-1*** **to the AWC sensed odor butanone.** “−” indicates unexposed animals and “+” indicates exposed animals. **Indicates *p*≤0.005 significant differences between chemotaxis index (CI) values between wildtype unexposed animals and *odr-1* or *daf-11* mutant unexposed animals. *Indicates *p*≤0.05 significant differences between wildtype odor-exposed CI values and *pde* quadruple mutant odor-exposed CI values.
(TIF)

Figure S2 **Chemotaxis response of wildtype, PDE quadruple mutants, and PDE quadruple mutants expressing rescuing** ***pde*** **transgenes to the AWC sensed odor benzaldehyde.** “−” indicates unexposed animals and “+” indicates exposed animals. *Indicates *p*≤0.05 significant differences between chemotaxis index values between PDE quadruple mutants expressing rescuing *pde* transgenes plus and minus odor.
(TIF)

Figure S3 **Populations of GFP::EGL-4 (** ***pyIs500*** **) expressing animals were incubated with 10 mM IBMX for 45 minutes and then exposed to the odor benzaldehyde with 10 mM IBMX.** The percentage of animals displaying nuclear GFP::EGL-4 were compared to populations exposed to odor alone for 80 minutes or populations exposed to no odor. *Indicates *p*≤0.05 significant differences.
(TIF)

Figure S4 **Chemotaxis responses of wildtype animals and the mutants,** ***py825*** **and** ***py827*** **to the AWC-sensed odor benzaldehyde.** “−” indicates unexposed animals and “+” indicates exposed animals. *indicates *p*≤0.05 significant differences between mutants and wildtype animals.
(TIF)

Figure S5 **PCR genotyping of the 397 bp deletion lesion of** ***py827*** **.** First lane: DNA marker; Lanes 2–4: *py827* gDNA PCR product; Lanes 5–7: N2 (wildtype) gDNA PCR product. For each template (*i.e*. *py827* and wildtype) the PCR reaction is performed in triplicate. Primer pairs used were: jz827-F GACTCCTTCTCAACTACCAGCTAAACAATG jz827-R CATCTGCGAGACGTACTGATAGAATACAAG.
(TIF)

Figure S6 **Image of a wildtype animal after 60 mins treatment with 100 mM EGTA.** The white dotted box indicates morphological defect in the fan-shaped AWC cilia after treatment.
(TIF)

Figure S7 **Image of the AWC neuron in a transgenic animal expressing RAB-8[Q67L] under an AWC promoter.** Expression of RAB-8[Q67L] in AWC causes defective cilia morphology.
(TIF)

Figure S8 **The AWC cilia surface area of adult animals grown at room temperature was calculated using Volocity® software.** Using the ‘measure objects’ tool the region of interest (ROI) was captured as indicated in image by a blue box and measured (µm^2^).
(TIF)

Table S1 **The AWC cilia surface area of adult animals grown at room temperature was calculated using Volocity® software.** The most distal (anterior) 5 µm region was selected as the region of interest (ROI) and this area was measured (µm^2^) using the ‘measure objects’ tool in Volocity®. Refer to Figure S8 for image on ROI capture. S.D. = standard deviation. *Indicates *p*≤0.05 significant differences compared with wildtype animals using a two-tailed Student t-test.
(DOC)

## References

[1] Calvert PD, Strissel KJ, Schiesser WE, Pugh EN, Arshavsky VY (2006). Light-driven translocation of signaling proteins in vertebrate photoreceptors.. Trends Cell Biol.

[2] Colbert HA, Bargmann CI (1995). Odorant-specific adaptation pathways generate olfactory plasticity in C. elegans.. Neuron.

[3] Ardiel EL, Rankin CH (2008). Behavioral Plasticity in the C. elegans Mechanosensory Circuit.. J Neurogenet.

[4] Biron D, Shibuya M, Gabel C, Wasserman SM, Clark DA (2006). A diacylglycerol kinase modulates long-term thermotactic behavioral plasticity in C. elegans.. Nat Neurosci.

[5] Kraus N, Chandrasekaran B (2010). Music training for the development of auditory skills.. Nat Rev Neurosci.

[6] Bargmann CI, Horvitz HR (1991). Chemosensory neurons with overlapping functions direct chemotaxis to multiple chemicals in C. elegans.. Neuron.

[7] Bargmann CI, Hartwieg E, Horvitz HR (1993). Odorant-selective genes and neurons mediate olfaction in C. elegans.. Cell.

[8] Sengupta P, Chou JH, Bargmann CI (1996). odr-10 encodes a seven transmembrane domain olfactory receptor required for responses to the odorant diacetyl.. Cell.

[9] Troemel ER, Chou JH, Dwyer ND, Colbert HA, Bargmann CI (1995). Divergent seven transmembrane receptors are candidate chemosensory receptors in C. elegans.. Cell.

[10] Lans H, Rademakers S, Jansen G (2004). A network of stimulatory and inhibitory Galpha-subunits regulates olfaction in Caenorhabditis elegans.. Genetics.

[11] Roayaie K, Crump JG, Sagasti A, Bargmann CI (1998). The G alpha protein ODR-3 mediates olfactory and nociceptive function and controls cilium morphogenesis in C. elegans olfactory neurons.. Neuron.

[12] Jansen G, Thijssen KL, Werner P, van der Horst M, Hazendonk E (1999). The complete family of genes encoding G proteins of Caenorhabditis elegans.. Nat Genet.

[13] Birnby DA, Link EM, Vowels JJ, Tian H, Colacurcio PL (2000). A transmembrane guanylyl cyclase (DAF-11) and Hsp90 (DAF-21) regulate a common set of chemosensory behaviors in caenorhabditis elegans.. Genetics.

[14] L'Etoile ND, Bargmann CI (2000). Olfaction and odor discrimination are mediated by the C. elegans guanylyl cyclase ODR-1.. Neuron.

[15] Coburn CM, Bargmann CI (1996). A putative cyclic nucleotide-gated channel is required for sensory development and function in C. elegans.. Neuron.

[16] Komatsu H, Mori I, Rhee JS, Akaike N, Ohshima Y (1996). Mutations in a cyclic nucleotide-gated channel lead to abnormal thermosensation and chemosensation in C. elegans.. Neuron.

[17] Chalasani SH, Chronis N, Tsunozaki M, Gray JM, Ramot D (2007). Dissecting a circuit for olfactory behaviour in Caenorhabditis elegans.. Nature.

[18] Luo DG, Xue T, Yau KW (2008). How vision begins: an odyssey.. Proc Natl Acad Sci U S A.

[19] Fu Y, Yau KW (2007). Phototransduction in mouse rods and cones.. Pflugers Arch.

[20] L'Etoile ND, Coburn CM, Eastham J, Kistler A, Gallegos G (2002). The cyclic GMP-dependent protein kinase EGL-4 regulates olfactory adaptation in C. elegans.. Neuron.

[21] Matsuki M, Kunitomo H, Iino Y (2006). Goalpha regulates olfactory adaptation by antagonizing Gqalpha-DAG signaling in Caenorhabditis elegans.. Proc Natl Acad Sci U S A.

[22] Nuttley WM, Atkinson-Leadbeater KP, Van Der Kooy D (2002). Serotonin mediates food-odor associative learning in the nematode Caenorhabditiselegans.. Proc Natl Acad Sci U S A.

[23] Kaye JA, Rose NC, Goldsworthy B, Goga A, L'Etoile ND (2009). A 3′UTR pumilio-binding element directs translational activation in olfactory sensory neurons.. Neuron.

[24] O'Halloran DM, Altshuler-Keylin S, Lee JI, L'Etoile ND (2009). Regulators of AWC-mediated olfactory plasticity in Caenorhabditis elegans.. PLoS Genet.

[25] Lee JI, O'Halloran DM, Eastham-Anderson J, Juang BT, Kaye JA (2010). Nuclear entry of a cGMP-dependent kinase converts transient into long-lasting olfactory adaptation.. Proc Natl Acad Sci U S A.

[26] Liu J, Ward A, Gao J, Dong Y, Nishio N (2010). C. elegans phototransduction requires a G protein-dependent cGMP pathway and a taste receptor homolog.. Nat Neurosci.

[27] Johnson JL, Leroux MR (2010). cAMP and cGMP signaling: sensory systems with prokaryotic roots adopted by eukaryotic cilia.. Trends Cell Biol.

[28] Ben-Shahar Y, Leung HT, Pak WL, Sokolowski MB, Robinson GE (2003). cGMP-dependent changes in phototaxis: a possible role for the foraging gene in honey bee division of labor.. J Exp Biol.

[29] Lucas C, Sokolowski MB (2009). Molecular basis for changes in behavioral state in ant social behaviors.. Proc Natl Acad Sci U S A.

[30] Fitzpatrick MJ, Feder E, Rowe L, Sokolowski MB (2007). Maintaining a behaviour polymorphism by frequency-dependent selection on a single gene.. Nature.

[31] Hong RL, Witte H, Sommer RJ (2008). Natural variation in Pristionchus pacificus insect pheromone attraction involves the protein kinase EGL-4.. Proc Natl Acad Sci U S A.

[32] Ortiz CO, Etchberger JF, Posy SL, Frokjaer-Jensen C, Lockery S (2006). Searching for neuronal left/right asymmetry: genomewide analysis of nematode receptor-type guanylyl cyclases.. Genetics.

[33] Perkins LA, Hedgecock EM, Thomson JN, Culotti JG (1986). Mutant sensory cilia in the nematode Caenorhabditis elegans.. Dev Biol.

[34] Signor D, Wedaman KP, Orozco JT, Dwyer ND, Bargmann CI (1999). Role of a class DHC1b dynein in retrograde transport of IFT motors and IFT raft particles along cilia, but not dendrites, in chemosensory neurons of living Caenorhabditis elegans.. J Cell Biol.

[35] Signor D, Rose LS, Scholey JM (2000). Analysis of the roles of kinesin and dynein motors in microtubule-based transport in the Caenorhabditis elegans nervous system.. Methods.

[36] Bailey CH, Kandel ER (1993). Structural changes accompanying memory storage.. Annu Rev Physiol.

[37] Moser MB, Trommald M, Andersen P (1994). An increase in dendritic spine density on hippocampal CA1 pyramidal cells following spatial learning in adult rats suggests the formation of new synapses.. Proc Natl Acad Sci U S A.

[38] Lamprecht R, LeDoux J (2004). Structural plasticity and memory.. Nat Rev Neurosci.

[39] Matsuzaki M, Honkura N, Ellis-Davies GC, Kasai H (2004). Structural basis of long-term potentiation in single dendritic spines.. Nature.

[40] Hallows JL, Chen K, DePinho RA, Vincent I (2003). Decreased cyclin-dependent kinase 5 (cdk5) activity is accompanied by redistribution of cdk5 and cytoskeletal proteins and increased cytoskeletal protein phosphorylation in p35 null mice.. J Neurosci.

[41] Lindsay RM, Wiegand SJ, Altar CA, DiStefano PS (1994). Neurotrophic factors: from molecule to man.. Trends Neurosci.

[42] Meng Y, Zhang Y, Tregoubov V, Janus C, Cruz L (2002). Abnormal spine morphology and enhanced LTP in LIMK-1 knockout mice.. Neuron.

[43] Corbin JD, Doskeland SO (1983). Studies of two different intrachain cGMP-binding sites of cGMP-dependent protein kinase.. J Biol Chem.

[44] Mukhopadhyay S, Lu Y, Shaham S, Sengupta P (2008). Sensory signaling-dependent remodeling of olfactory cilia architecture in C. elegans.. Dev Cell.

[45] Orozco JT, Wedaman KP, Signor D, Brown H, Rose L (1999). Movement of motor and cargo along cilia.. Nature.

[46] Ou G, Blacque OE, Snow JJ, Leroux MR, Scholey JM (2005). Functional coordination of intraflagellar transport motors.. Nature.

[47] Evans JE, Snow JJ, Gunnarson AL, Ou G, Stahlberg H (2006). Functional modulation of IFT kinesins extends the sensory repertoire of ciliated neurons in Caenorhabditis elegans.. J Cell Biol.

[48] Blacque OE, Perens EA, Boroevich KA, Inglis PN, Li C (2005). Functional genomics of the cilium, a sensory organelle.. Curr Biol.

[49] Blacque OE, Leroux MR (2006). Bardet-Biedl syndrome: an emerging pathomechanism of intracellular transport.. Cell Mol Life Sci.

[50] Kim JC, Badano JL, Sibold S, Esmail MA, Hill J (2004). The Bardet-Biedl protein BBS4 targets cargo to the pericentriolar region and is required for microtubule anchoring and cell cycle progression.. Nat Genet.

[51] Nausch LW, Ledoux J, Bonev AD, Nelson MT, Dostmann WR (2008). Differential patterning of cGMP in vascular smooth muscle cells revealed by single GFP-linked biosensors.. Proc Natl Acad Sci U S A.

[52] Isner JC, Maathuis FJ (2011). Measurement of cellular cGMP in plant cells and tissues using the endogenous fluorescent reporter FlincG.. Plant J.

[53] Brenner S (1974). The genetics of Caenorhabditis elegans.. Genetics.

[54] Hobert O (2002). PCR fusion-based approach to create reporter gene constructs for expression analysis in transgenic C. elegans.. Biotechniques.

