# Supplementary figures and images for: Changes in cGMP Levels Affect the Localization of EGL-4 in AWC in Caenorhabditis elegans
Source: PLoS One. 2012 Feb 3;7(2):e31614. doi: 10.1371/journal.pone.0031614 (PMC3272044; doi:10.1371/journal.pone.0031614)

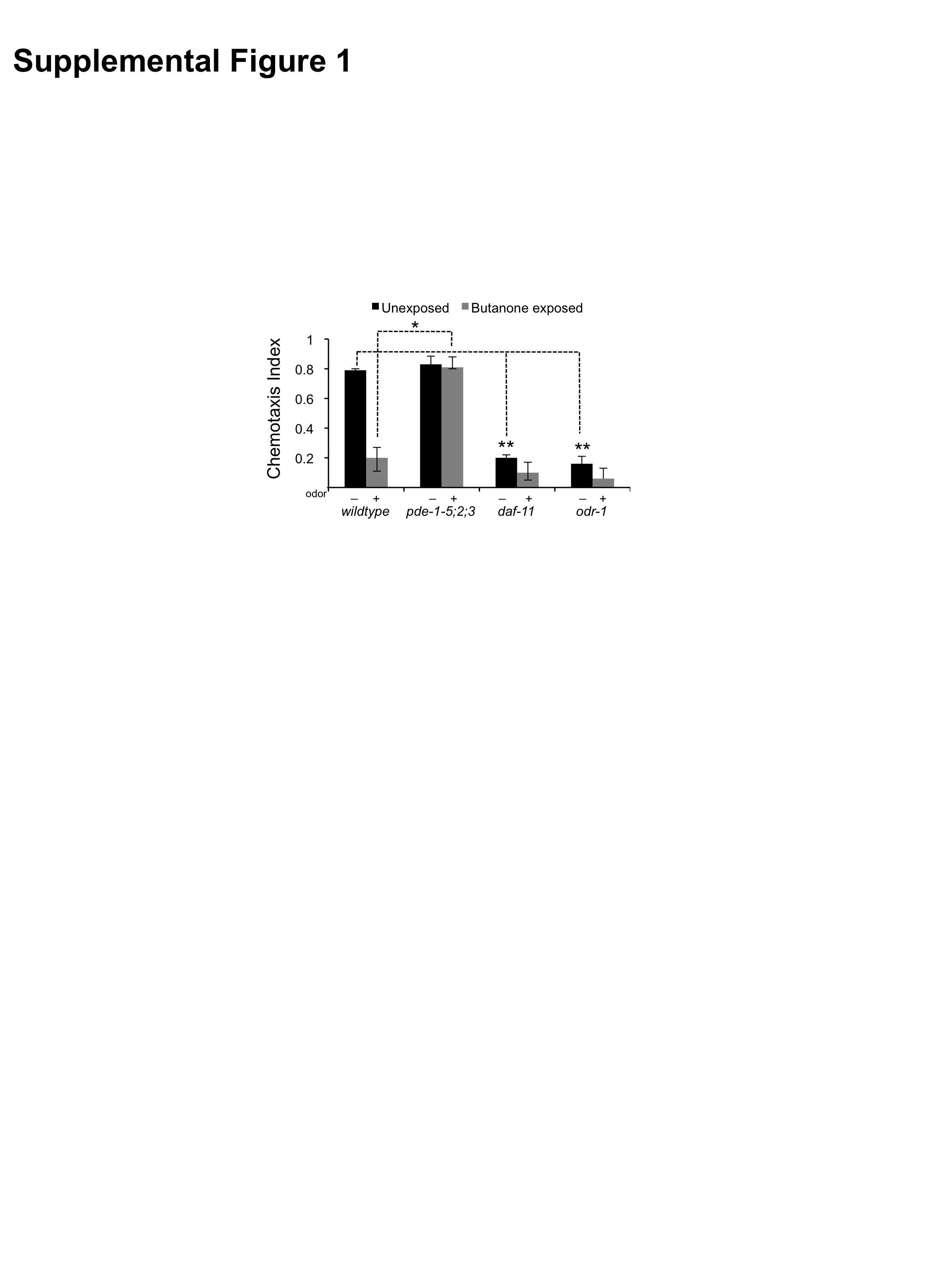

Supplement: Figure S1 — Chemotaxis response of the PDE quadruple mutant and the guanylyl cyclase mutants daf-11 and odr-1 to the AWC sensed odor butanone. “−” indicates unexposed animals and “+” indicates exposed animals. **Indicates p≤0.005 significant differences between chemotaxis index (CI) values between wildtype unexposed animals and odr-1 or daf-11 mutant unexposed animals. *Indicates p≤0.05 significant differences between wildtype odor-exposed CI values and pde quadruple mutant odor-exposed CI values. (TIF) [file pone.0031614.s001.tif]

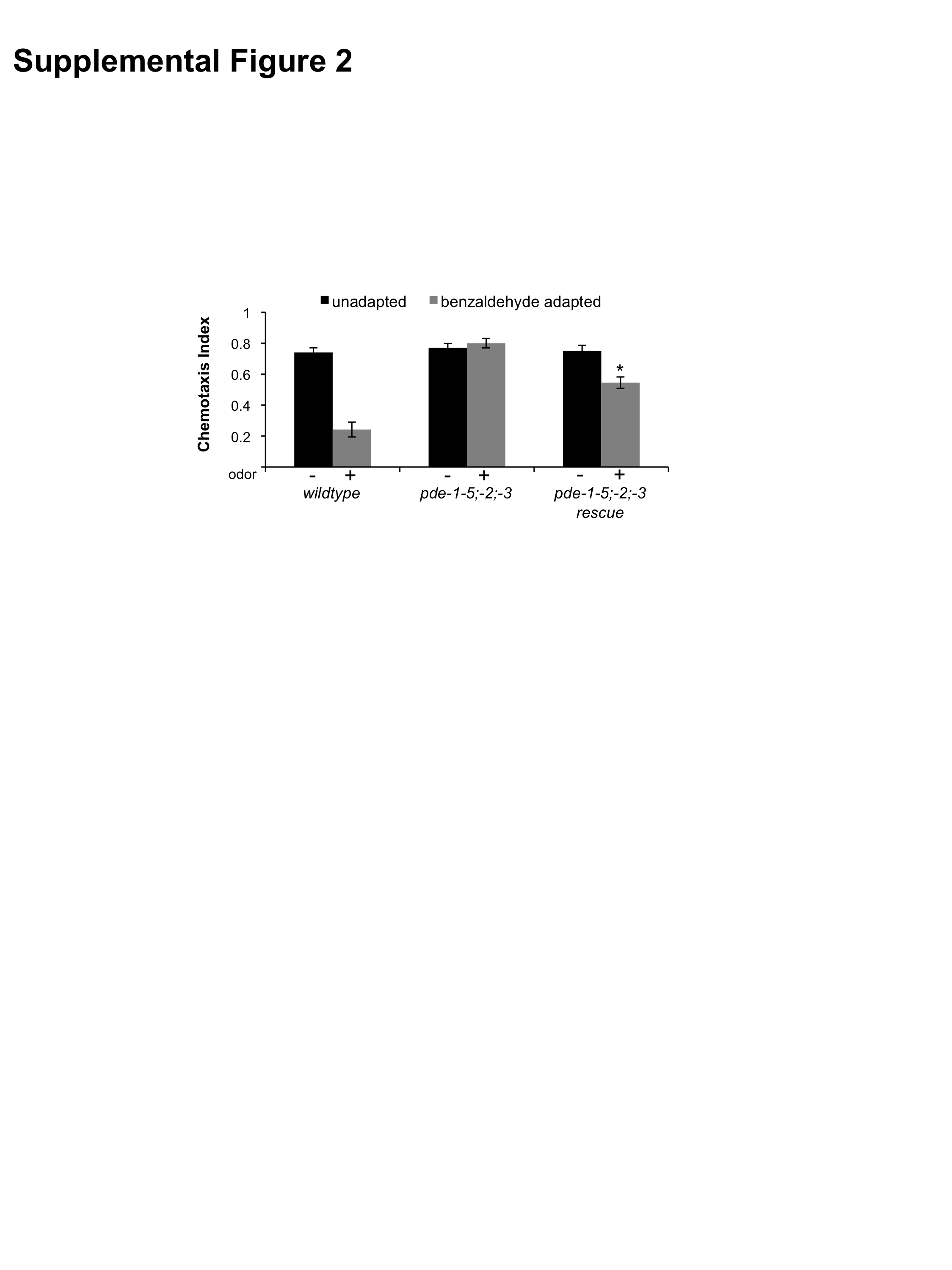

Supplement: Figure S2 — Chemotaxis response of wildtype, PDE quadruple mutants, and PDE quadruple mutants expressing rescuing pde transgenes to the AWC sensed odor benzaldehyde. “−” indicates unexposed animals and “+” indicates exposed animals. *Indicates p≤0.05 significant differences between chemotaxis index values between PDE quadruple mutants expressing rescuing pde transgenes plus and minus odor. (TIF) [file pone.0031614.s002.tif]

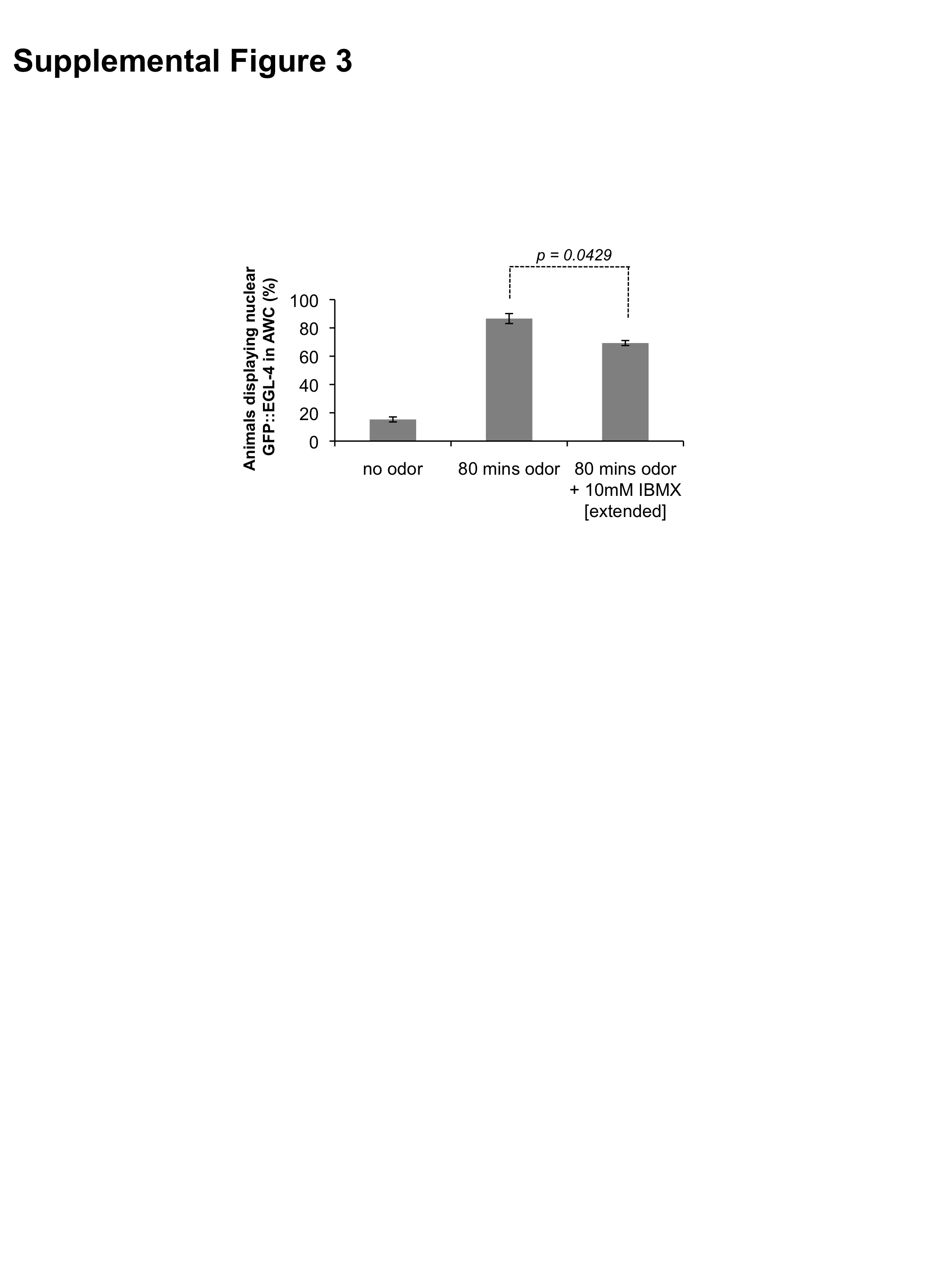

Supplement: Figure S3 — Populations of GFP::EGL-4 ( pyIs500 ) expressing animals were incubated with 10 mM IBMX for 45 minutes and then exposed to the odor benzaldehyde with 10 mM IBMX. The percentage of animals displaying nuclear GFP::EGL-4 were compared to populations exposed to odor alone for 80 minutes or populations exposed to no odor. *Indicates p≤0.05 significant differences. (TIF) [file pone.0031614.s003.tif]

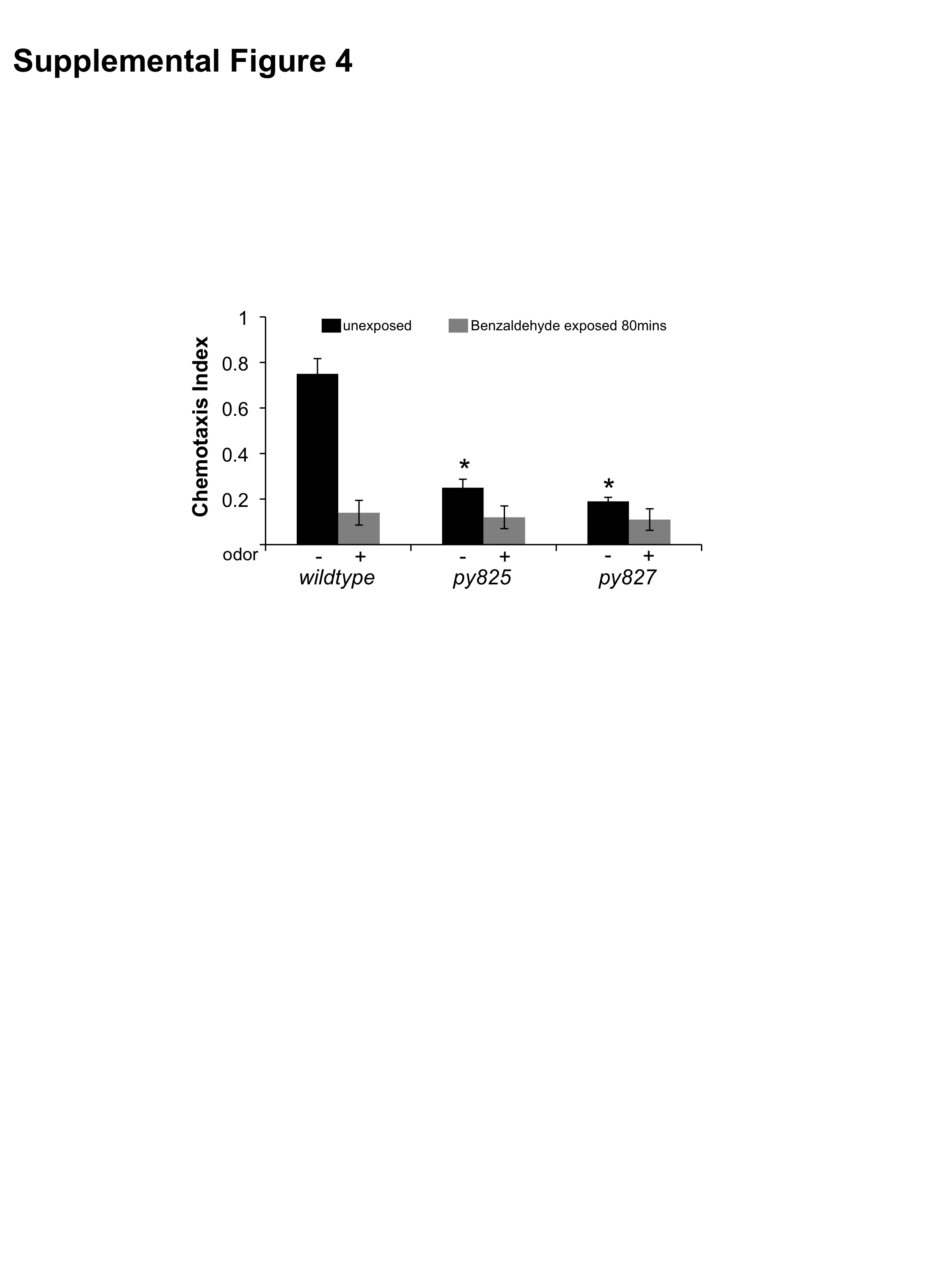

Supplement: Figure S4 — Chemotaxis responses of wildtype animals and the mutants, py825 and py827 to the AWC-sensed odor benzaldehyde. “−” indicates unexposed animals and “+” indicates exposed animals. *indicates p≤0.05 significant differences between mutants and wildtype animals. (TIF) [file pone.0031614.s004.tif]

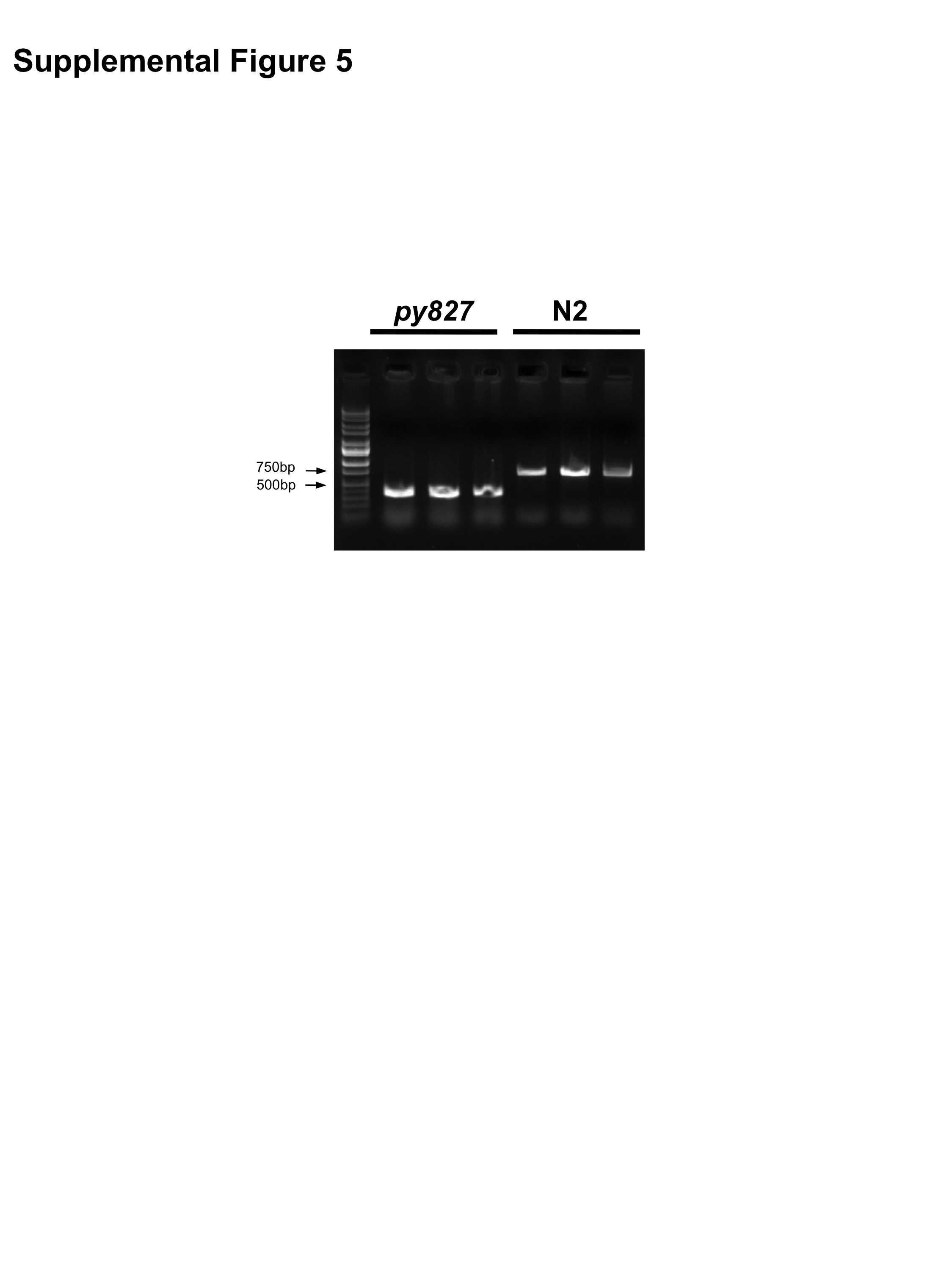

Supplement: Figure S5 — PCR genotyping of the 397 bp deletion lesion of py827 . First lane: DNA marker; Lanes 2–4: py827 gDNA PCR product; Lanes 5–7: N2 (wildtype) gDNA PCR product. For each template (i.e. py827 and wildtype) the PCR reaction is performed in triplicate. Primer pairs used were: jz827-F GACTCCTTCTCAACTACCAGCTAAACAATG jz827-R CATCTGCGAGACGTACTGATAGAATACAAG. (TIF) [file pone.0031614.s005.tif]

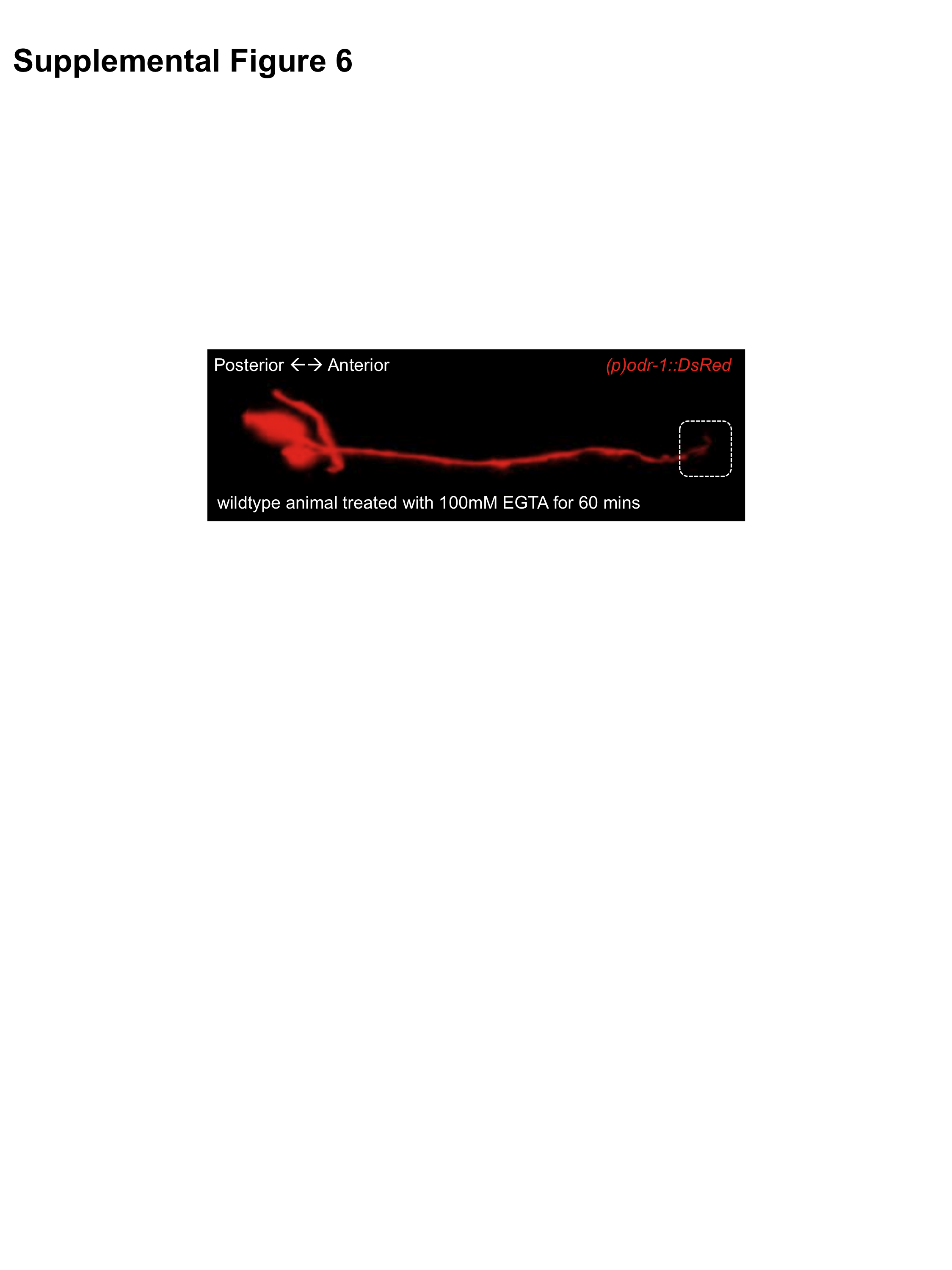

Supplement: Figure S6 — Image of a wildtype animal after 60 mins treatment with 100 mM EGTA. The white dotted box indicates morphological defect in the fan-shaped AWC cilia after treatment. (TIF) [file pone.0031614.s006.tif]

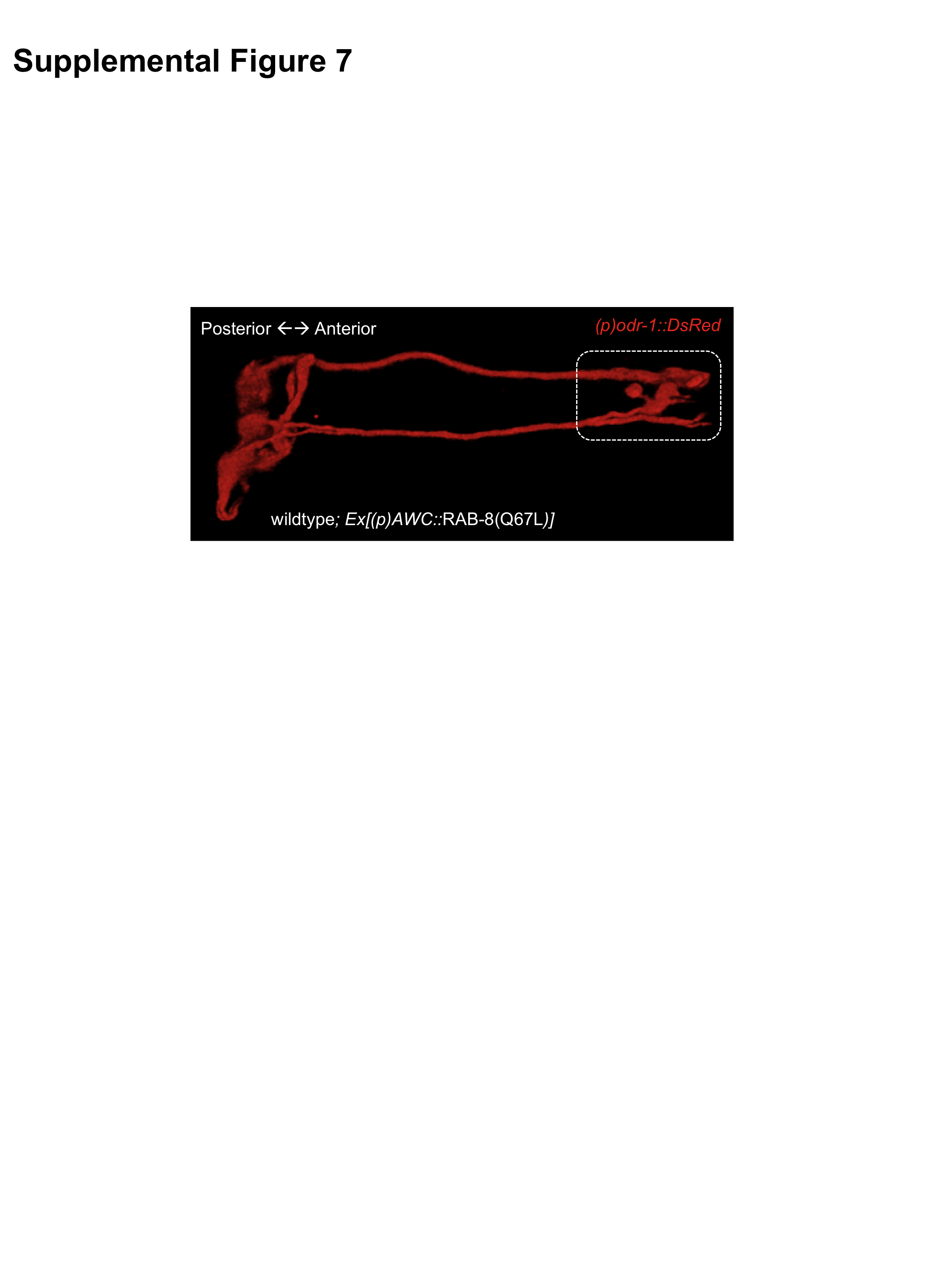

Supplement: Figure S7 — Image of the AWC neuron in a transgenic animal expressing RAB-8[Q67L] under an AWC promoter. Expression of RAB-8[Q67L] in AWC causes defective cilia morphology. (TIF) [file pone.0031614.s007.tif]

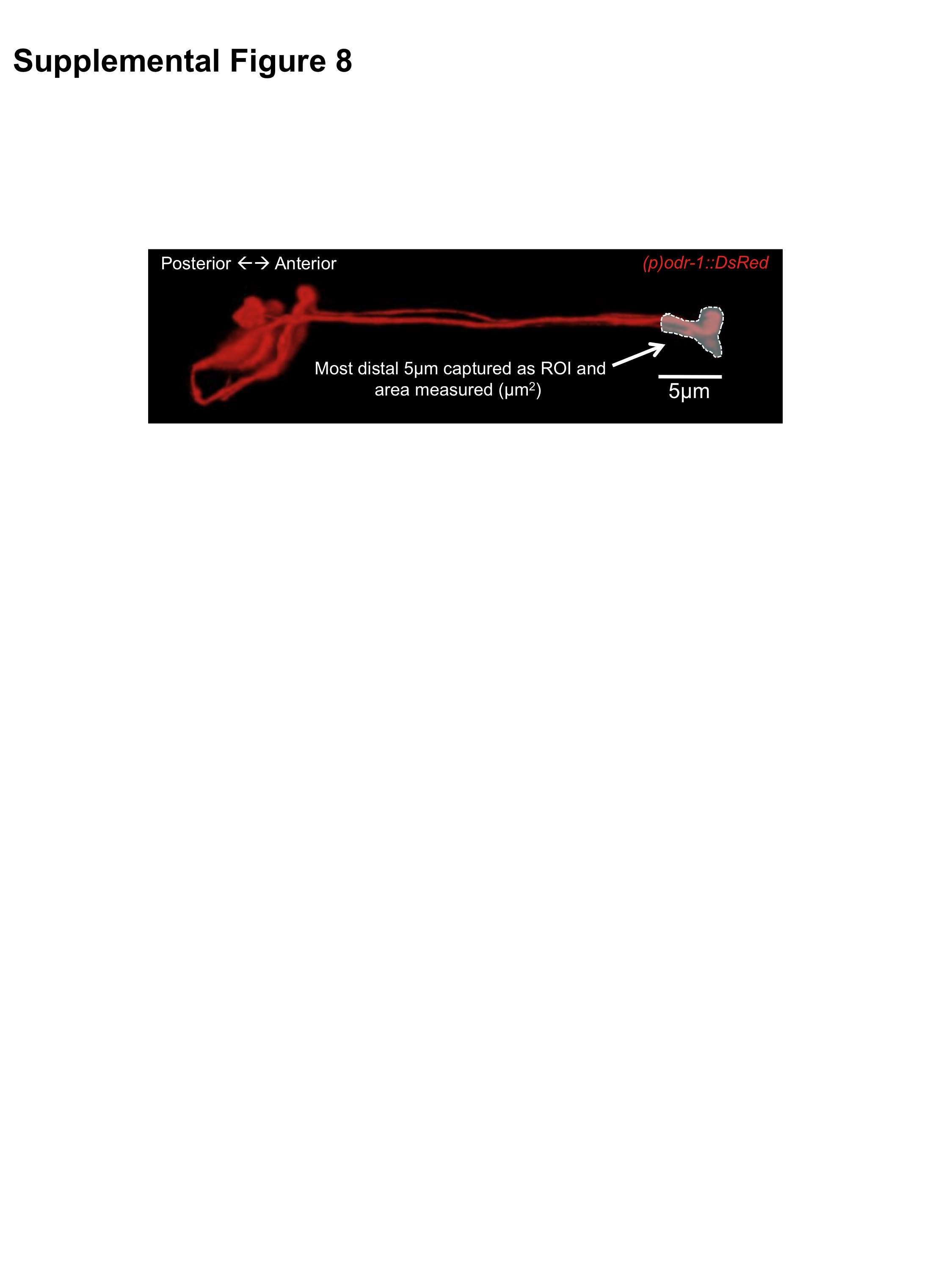

Supplement: Figure S8 — The AWC cilia surface area of adult animals grown at room temperature was calculated using Volocity® software. Using the ‘measure objects’ tool the region of interest (ROI) was captured as indicated in image by a blue box and measured (µm2). (TIF) [file pone.0031614.s008.tif]
