# Supplementary material for: Changes in cGMP Levels Affect the Localization of EGL-4 in AWC in Caenorhabditis elegans
Source: PLoS One. 2012 Feb 3;7(2):e31614. doi: 10.1371/journal.pone.0031614 (PMC3272044; doi:10.1371/journal.pone.0031614)
Supplement: Table S1 — The AWC cilia surface area of adult animals grown at room temperature was calculated using Volocity® software. The most distal (anterior) 5 µm region was selected as the region of interest (ROI) and this area was measured (µm2) using the ‘measure objects’ tool in Volocity®. Refer to Figure S8 for image on ROI capture. S.D. = standard deviation. *Indicates p≤0.05 significant differences compared with wildtype animals using a two-tailed Student t-test. (DOC) [file pone.0031614.s009.doc]

Table S1. Surface Area of AWC Cilia.

| **Strain** | **Cilia Surface Area (μm^2^) ± S.D.** |
| --- | --- |
| wildtype | 11.6 ± 1.14 |
| wildtype; Ex[(p)*odr-3*::NLS::GFP::EGL-4] | 12.1 ± 1.22 |
| *egl-4(n479)*; Ex[(p)*odr-3*::GFP::ΔNLS EGL-4] | 13.16 ± 0.8 |
| *osm-1(py825)* | 7.44 ± 0.6* |
| *che-3(py827)* | 8.05 ± 0.47* |
| *osm-1(py825)*; Ex[(p)*odr-3*::GFP::ΔNLS EGL-4] | 7.49 ± 0.36* |
